# Drivers and Annual Totals of Methane Emissions From Dutch Peatlands

**DOI:** 10.1111/gcb.17590

**Published:** 2024-12-06

**Authors:** Alexander J. V. Buzacott, Bart Kruijt, Laurent Bataille, Quint van Giersbergen, Tom S. Heuts, Christian Fritz, Reinder Nouta, Gilles Erkens, Jim Boonman, Merit van den Berg, Jacobus van Huissteden, Ype van der Velde

**Affiliations:** ^1^ Earth and Climate Vrije Universiteit Amsterdam Amsterdam Netherlands; ^2^ Water Systems and Global Change Group Wageningen University Wageningen Netherlands; ^3^ Radboud Institute for Biological and Environmental Sciences Radboud University Nijmegen Netherlands; ^4^ Wetterskip Fryslân Leeuwarden Netherlands; ^5^ Deltares Research Institute Utrecht Netherlands; ^6^ Department of Physical Geography Utrecht University Utrecht Netherlands; ^7^ VOF Kytalyk Carbon Cycle Research Epse Netherlands

**Keywords:** CH_4_, eddy covariance, flux driver, greenhouse gas, land use change, machine learning, rewet

## Abstract

Rewetting peatlands is required to limit carbon dioxide (CO_2_) emissions, however, raising the groundwater level (GWL) will strongly increase the chance of methane (CH_4_) emissions which has a higher radiative forcing than CO_2_. Data sets of CH_4_ from different rewetting strategies and natural systems are scarce, and quantification and an understanding of the main drivers of CH_4_ emissions are needed to make effective peatland rewetting decisions. We present a large data set of CH_4_ fluxes (FCH_4_) measured across 16 sites with eddy covariance on Dutch peatlands. Sites were classified into six land uses, which also determined their vegetation and GWL range. We investigated the principal drivers of emissions and gapfilled the data using machine learning (ML) to derive annual totals. In addition, Shapley values were used to understand the importance of drivers to ML model predictions. The data showed the typical controls of FCH_4_ where temperature and the GWL were the dominant factors, however, some relationships were dependent on land use and the vegetation present. There was a clear average increase in FCH_4_ with increasing GWLs, with the highest emissions occurring at GWLs near the surface. Soil temperature was the single most important predictor for ML gapfilling but the Shapley values revealed the multi‐driver dependency of FCH_4_. Mean annual FCH_4_ totals across all land uses ranged from 90 _±_ 11 to 632 _±_ 65 kg CH_4_ ha^−1^ year^−1^ and were on average highest for semi‐natural land uses, followed by paludiculture, lake, wet grassland and pasture with water infiltration system. The mean annual flux was strongly correlated with the mean annual GWL (*R* ^2^ = 0.80). The greenhouse gas balance of our sites still needs to be estimated to determine the net climate impact, however, our results indicate that considerable rates of CO_2_ uptake and long‐term storage are required to fully offset the emissions of CH_4_ from land uses with high GWLs.

## Graphical abstract

Drained peatlands areas need to raise the groundwater level to limit emissions of carbon dioxide and to reduce land subsidence. A concern upon raising the groundwater level in formerly drained peatlands is the emissions of methane, a stronger greenhouse gas than carbon dioxide. We explored emissions of methane from different land uses with different average annual groundwater levels across the coastal plain of the Netherlands. Land uses with higher groundwater levels had higher emissions of methane, with a near‐linear increase in annual emissions. A comparison of different greenhouse gas emissions with the groundwater levels remains to be done.

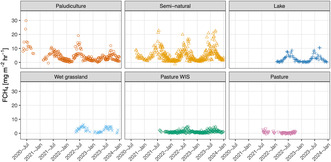

## 1 Introduction

Methane (CH_4_) is, after carbon dioxide (CO_2_), the second most important greenhouse gas globally. Its atmospheric concentration has risen from 729 ppb in 1750 to 1879 ppb in 2020, contributing 0.54 W m^–2^ and approximately 0.5ºC to climate warming (IPCC 2021; Lan et al. 2021). Natural wetlands and enteric fermentation and manure from agriculture contribute between 17%–21% and 13%–18%, respectively, to CH_4_ emissions for the temperate zone latitudinal band between 30° and 60° based on bottom‐up estimates (Saunois et al. 2020). A change in agricultural practice and management of natural wetlands contributes to a change in CH_4_ emission. CH_4_ has a much larger instantaneous radiative contribution to the warming of the atmosphere than CO_2_, but its lifetime of 8–12 years is much shorter (Stevenson et al. 2020; IPCC 2021). Due to the short lifetime, changes in CH_4_ emission have a relatively rapid effect on climate warming, whereas emitted CO_2_ has a long‐lasting effect.

Microbial oxidation of drained peatlands is a large source of CO_2_ globally at approximately 2 Gt year^–1^ and contributes around 5% of anthropogenic greenhouse gas (GHG) emissions annually (Joosten et al. 2016). Drainage of peatlands also causes land subsidence (Hoogland, van den Akker, and Brus 2012; Erkens, van der Meulen, and Middelkoop 2016) which can have large economic effects (van den Born et al. 2016). Rewetting peatlands is an often‐promoted measure to reduce the decomposition of organic matter and to potentially sequester new carbon (Zerbe et al. 2013; Wilson et al. 2016; Günther et al. 2020; Mrotzek et al. 2020; Schwieger et al. 2021). Various measures have been proposed to curb CO_2_ emission and land subsidence (e.g., Geurts et al. 2019; Kwakernaak et al. 2010; Querner et al. 2012; Fritz et al. 2014; Temmink et al. 2017; Vroom et al. 2018; Lahtinen et al. 2022), all directed at increasing the groundwater level in peatlands: (1) Rewetting by blocking of drainage ditches and change of land use towards (semi)natural peatlands with nature conservation/rewilding goals (e.g., Hendriks et al. 2007; Schrier‐Uijl et al. 2014). Although this may not return peatlands to their predrainage state, it reduces carbon emissions from peat soils (Kreyling et al. 2021). (2) (Partial) rewetting, in combination with growing crops that thrive under high groundwater level conditions (paludiculture), such as *Typha*, *Sphagnum*, cranberry and various other crops that produce substrate, energy source, isolation material or food (e.g., Lahtinen et al. 2022; Wichtmann, Schröder, and Joosten 2016; Ziegler et al. 2021), with the additional advantage that some crops can reduce the agricultural nutrient load of surface waters (Vroom et al. 2018). (3) (Partial) rewetting, in combination with adaptations in dairy farming or cattle breeding to wetter conditions, possibly in combination with paludiculture crops (Liu et al. 2022). (4) Technical measures that create a more homogeneous groundwater level in time and space in peatland parcels, for instance, by installing pipes that infiltrate water from ditches into the peat in dry periods and drain the peat in wet periods (mostly winter) (Querner, Jansen, and Kwakernaak 2008; Querner et al. 2012; van den Akker and Hendriks 2017; Hoekstra, van Schie, and van Hardeveld 2020; van Hardeveld et al. 2020). Various implementations have become known as “reversed drainage” or “water infiltration systems” (WIS).

Changes in the management of peatlands affect the emission of both CO_2_ and CH_4_. A concern upon rewetting disturbed peatlands are the emissions of CH_4_ (Günther et al. 2015) and it has been termed a “biogeochemical compromise” (Hemes et al. 2018). While studies have shown that rewetting reduces climate warming despite increases in CH_4_ emissions (e.g., Günther et al. 2020), emission responses to rewetting are not certain (Kreyling et al. 2021), and there is limited data on CH_4_ emissions for many rewetting strategies.

CH_4_ emission from soils and ecosystems is the result of complex interactions between soil hydrology, soil biogeochemistry and vegetation. Fundamentally, it is a balance between CH_4_ production by methanogenic archaea under strictly anaerobic conditions and CH_4_ oxidation to CO_2_ by methanotrophic bacteria in oxygenated parts of the soil and plant tissues (e.g., Bodelier and Steenbergh 2014; Walter and Heimann 2000; Olefeldt et al. 2013; van Huissteden 2020). This balance is strongly influenced by three transport pathways of CH_4_: diffusion through soil pores, transport by gas bubbles rising through saturated soil and water and transport by aerenchymous plant parts (hollow roots and stems of wetland plants). The latter two mechanisms bypass CH_4_ oxidation by methanotrophs in the soil matrix. Plant transport processes vary among plant species with differences in physiological characteristics (Vroom et al. 2022). Several plant species groups, in particular wetland mosses such as *Sphagnum*, depress CH_4_ emission by the symbiotic presence of methanotrophs inside leaves; the CO_2_ produced by the methanotrophs is used for photosynthesis by the moss (e.g., Kip et al. (2010)).

Vegetation also has an effect on CH_4_ emission by supplying methanogens with labile organic matter in the form of root exudates that serve as a substrate for methanogens (King and Reeburgh 2002; Bodelier and Steenbergh 2014; Waldo et al. 2019). Due to this interaction with soil chemistry, groundwater level and vegetation, CH_4_ emission in wetland environments and agricultural soil–plant systems has a very high spatial variability. Measurements in natural wetland systems show an order of magnitude variation in emission over short distances (van Huissteden, Maximov, and Dolman 2005; Hendriks, van Huissteden, and Dolman 2010). The relation with plant species composition is strong enough to serve as a predictor for the magnitude of CH_4_ fluxes (Dias et al. 2010; Couwenberg et al. 2011). In general, more nutrient‐rich systems produce higher fluxes than nutrient‐poor, ombrotrophic systems since nutrients boost the primary production of vegetation that supplies the substrate for the methanogens (Schrier‐Uijl et al. 2011; Bodelier 2011; Lara et al. 2019).

Approximately 90% of the remaining peat area in the Netherlands is drained and used for predominately dairy agriculture. The drainage of Dutch peatlands contributes approximately 3% of the total annual Dutch CO_2_ emissions (CBS 2023). Currently, these drained agricultural peatland soils are not a large source of CH_4_ and may even be a small sink, except in ditches, the border zones along ditches, or at short periods of high groundwater level (Schrier‐Uijl et al. 2010b; Vermaat et al. 2011; Schrier‐Uijl et al. 2014; Gremmen et al. 2022; Hendriks et al. 2024). However, raising the groundwater level, with and without changing the land use, potentially increases CH_4_ emission from soils substantially. Such an increase may be temporary or permanent (Abdalla et al. 2016). In the Netherlands and in other nations, there is a strong demand for CH_4_ emission data to construct greenhouse gas balances and to choose appropriate rewetting strategies due to the strong warming effect of CH_4_. The data are also urgently required to understand drivers of emissions to explain and predict emissions, especially for emission upscaling and to also guide management practices to potentially reduce emissions.

In this study, we used a network of eddy covariance systems to study the variability of CH_4_ emissions across different land uses, management practices and wetness conditions in nutrient‐rich peatlands on the coastal plain of the Netherlands. We report emissions from several wet and rewetted land uses, including paludiculture, semi‐natural systems, wet grasslands and pastures with water infiltration systems. The main aims were to (1) quantify and compare emission rates of CH_4_ across land uses, (2) explore key relationships between CH_4_ emissions and meteorological, soil and water characteristics and (3) construct annual CH_4_ flux totals to assess the net annual emission, inspect the overriding controls on annual emission and evaluate the emission totals in the context of peatland rewetting.

## 2 Materials and Methods

### 2.1 Study Area

**FIGURE 1:**
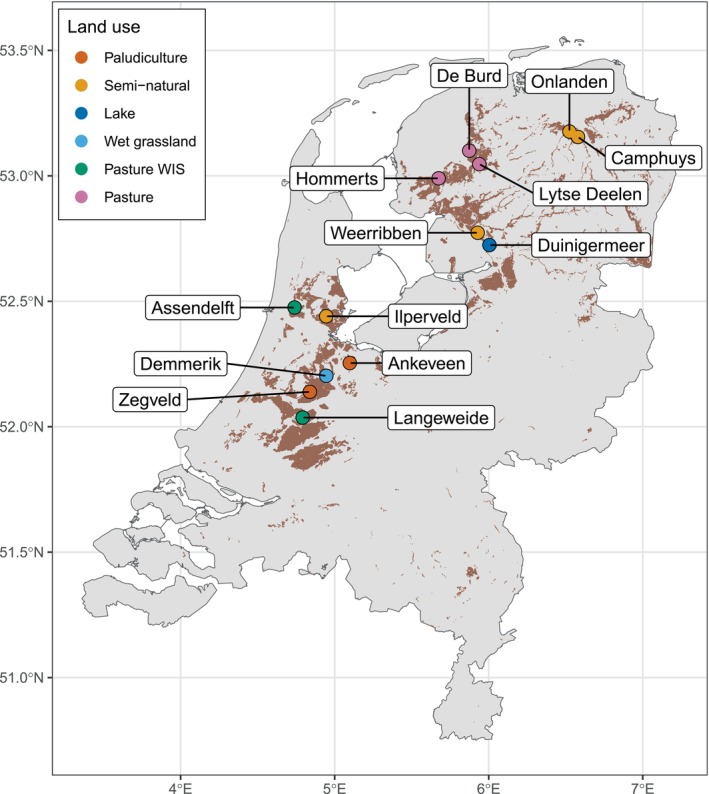
Location of field sites measuring methane fluxes across the Netherlands. The sites Hommerts, De Burd and Lytse Deelen have paired measurement plots. The brown shading indicates the current peatland extent. Map lines delineate study areas and do not necessarily depict accepted national boundaries.

Methane fluxes were measured on peatlands in the Netherlands (Figure 1). The Netherlands has a temperate humid climate, with a mean annual air temperature of 10.6 ± 0.7ºC between 1991 and 2020 (De Bilt, central Netherlands, KNMI ID: 260). The mean winter temperature is 3.9 ± 1.4ºC and the summer average is 17.4 ± 0.8ºC. The yearly average precipitation total over the same period is 855 ± 153 mm. The peatlands at the study sites are coastal fen peatlands (surface elevation below 1 m above mean sea level) formed on the landward side of tidal flats, estuarine and lagoonal sands and clays and behind a chain of Holocene coastal barrier islands. To the west and north and in the fluvial plains of the Scheldt, Meuse and Rhine, the peats are intercalated with the sands and clays. On the inland side, the peat deposits overlay Pleistocene glacial, fluvial and aeolian deposits. Outside the coastal plain on the higher Pleistocene surfaces, large areas were also covered by peat as fens in smaller river valleys and as bogs on poorly drained flat areas.

A total of 16 unique eddy covariance (EC) measurement sites were included in our analysis which consisted of eight permanent and eight “mobile” installations (Tables 1, 2). Mobile installations are a set of EC masts that are rotated between sites. One EC system was shared between Onlanden and Camphuys between July 2020 and until October 2023, after which the EC system remained at Onlanden. At Hommerts, De Burd and Lytse Deelen, there are two measurement parcels at each site and two EC systems were rotated through these three sites. The measurement sites were categorised into six groups based on the main land use type and groundwater levels: paludiculture, semi‐natural, lake, wet grassland, pastures with water infiltration systems (WIS) and pasture. The vegetation is determined by land use and the nonagricultural sites had a mixture of species present (Table 1). The paludiculture site Zegveld is vegetated by *Typha latifolia* (broadleaf cattail) and the Ankeveen site has a mixture of *Typha angustifolia* (narrowleaf cattail), *Carex* (sedges), *Juncus* (rushes), *T. latifolia* in the measurement area. The semi‐natural wetlands are largely a result of rewetting of former agricultural land and are dominated by *Phragmites australis* , *Carex* spp. and *Sparganium erectum* . The lake site was vegetated by *Potamogeton* (pondweed) species and helophytes (*Phragmites* and *Typha*) on the lake edge. The one wet grassland site, Demmerik, has more diverse grass species and functions as a bird meadow but it is grazed with low intensity by cattle during the summer. Agricultural land use is represented by pastures dominated by *Lolium perenne* (perennial ryegrass).

**TABLE 1:** General information on the sites included in this study.

Site	Land use	Lat (º)	Lon (º)	Elev (m)	Soil	Peat thickness (m)	Vegetation	Date range	Mean annual GWL (cm)	Mean summer GWL (cm)
Ankeveen	Paludiculture	52.25	5.10	−1.3	Peaty sand	0.2	*T. angustifolia, Carex* spp., *Juncus spp., T. latifolia*	2021 Jul 2023 Dec	−4	−12
Zegveld	Paludiculture	52.14	4.84	−2.4	Peat	6.0	*T. latifolia*	2020 May–2023 Dec	8	5
Camphuys	Semi‐natural	53.15	6.58	0.6	Peat	2.5	*S. erectum, G* *. maxima* , *P* *. australis*	2020 Sep–2023 Oct	−2	−10
Ilperveld	Semi‐natural	52.44	4.95	−0.5	Peat	3.0	*Juncus spp., P. australis, Sphagnum* spp.	2021 Aug–2023 Dec	−3	−6
Onlanden	Semi‐natural	53.18	6.52	−1.8	Peat	3.0	*T. latifolia, P. australis*	2020 Sep–2023 Dec	8	−3
Weerribben	Semi‐natural	52.77	5.93	−0.6	Peat	2.3	*P. australis, Sphagnum* spp.	2021 Aug ‐ 2023 Dec	−8	−18
Duinigermeer	Lake	52.72	6.00	−0.5	Organic sediment		*Potamogeton* spp.	2021 Dec–2023 Dec		
Demmerik	Wet grassland	52.20	4.95	−2.0	Peat	6.0	*Alopecurus* spp., *Poa pratensis* , *Danthonia decumbens*	2021 Jun–2023 Dec	−21	−27
Assendelft	Pasture WIS	52.48	4.74	−1.8	Peat	2.0	*L. perenne*	2021 Aug–2023 Dec	−24	−34
Langeweide	Pasture WIS	52.04	4.79	−2.0	Peat	7.2	*L. perenne*	2022 Mar–2023 Dec	−29	−51
Hommerts C	Pasture	52.99	5.68	−0.9	Peat	2.4	*L. perenne*	2021 Aug–2022 Sep	−55	−77
Hommerts H	Pasture	52.99	5.67	−1.0	Peat	2.3	*L. perenne*	2021 Aug–2022 Sep	−45	−61
Lytse Deelen C	Pasture	53.05	5.94	−0.9	Peat	1.6	*L. perenne*	2021 Jul –2022 Sep	−61	−94
Lytse Deelen H	Pasture	53.05	5.94	−1.0	Peat	1.8	*L. perenne*	2021 Jul –2022 Sep	−35	−51
De Burd C	Pasture	53.10	5.87	−1.2	Peat	2.7	*L. perenne*	2021 Jul –2022 Sep	−44	−63
De Burd T	Pasture	53.10	5.86	−0.9	Peat	1.5	*L. perenne*	2021 Jul –2022 Sep	−52	−78

*Note:* More comprehensive soil information is presented in Table S1.1. Groundwater‐level (GWL) values are positive above the surface and negative below.

**TABLE 2:** Eddy covariance information for sites included in this study.

Site	Installation	Measurement height (m)	Canopy height (m)	u* threshold (m s^–1^)	Min target area in FP (%)	Target area in mean FP (%)	Open water in mean FP (%)	FCH_4_, *n* (%)
Ankeveen	Permanent	2.5–5*	0.3–3.0	0.08	50	74	2.3	10,944 (25%)
Zegveld	Permanent	3.0	0.3–2.0	0.06	70	78	1.6	5273 (8%)
Camphuys	Mobile	4.8–6.1*	2.0	0.15	50	83	5.0	8908 (16%)
Ilperveld	Permanent	2.5	0.1–0.5	0.14	50	62	38.0	6130 (15%)
Onlanden	Mobile	3.7–5.8*	1.0–2.0	0.14	50	99	14.0	9646 (16%)
Weerribben	Permanent	2.5, 6*	0.2–1.5	0.09	50	71	8.0	13,909 (33%)
Duinigermeer	Permanent	2.5	0	0.05	70	92	92.0	10,408 (29%)
Demmerik	Permanent	6.8	0.2	0.11	50	70	24.0	5115 (18%)
Assendelft	Permanent	2.5	0.2	0.10	50	78	1.0	10,785 (26%)
Langeweide	Permanent	6.6	0.2	0.17	50	81	16.0	8381 (23%)
Hommerts C	Mobile	1.8	0.2	0.10	50	87	3.0	261 (1%)
Hommerts H	Mobile	1.8	0.2	0.11	50	89	2.0	194 (1%)
Lytse Deelen C	Mobile	1.8	0.2	0.10	50	82	3.0	181 (1%)
Lytse Deelen H	Mobile	1.8	0.2	0.12	50	88	4.0	326 (2%)
De Burd C	Mobile	1.8	0.2	0.10	50	89	3.0	337 (2%)
De Burd T	Mobile	1.8	0.2	0.12	50	87	3.0	313 (1%)

*Notes:* The installation status refers to whether a tower was fixed (permanent) or if it was part of a rotating set (mobile). The minimum target area in the footprint (FP) refers to the minimum target source area threshold for a flux timestep to be accepted for a site. The mean target area FP is the average after the minimum threshold filtering. The open water in the mean FP was estimated using the weighted area of open water features in the mean FP climatologies. The amount of half‐hourly measurements of methane fluxes (FCH_4_) is shown in the last column. An asterisk indicates that the measurement height varied over time depending on vegetation height or desire to increase the fetch. Mean FP climatologies are presented in Figure S1.1–S1.13, and more detailed site‐year gap statistics are presented in Table S1.2.

All of our study sites are located on fen peat with eutrophic/nutrient rich vegetation. More detailed soil profile information for nearly all study sites is provided in Table S1.1, including soil class and texture, botanical origin, decomposition state, carbon and nitrogen content. A reference profile from a nearby site, Aldeboarn (Aben et al. 2024), was provided as a reference for the sites De Burd, Hommerts and Lytse Deelen. Sediment information was not available for Duinigermeer. The porewater at the study sites was fresh (electroconductivity < 1500 μS cm^–1^) for all sites except Assendelft and Ilperveld which had slightly brackish water with mean electroconductivity values of 2295 and 2510 μS cm^–1^, respectively. These sites are the closest to the coast with distances of approximately 11 and 25 km, respectively, where the higher salinity is caused by upward seepage. Pasture and pasture WIS sites were fertilised using manure slurry. Estimated mean annual application rates of slurry were 1.1 t C ha^–1^ at Assendelft, 2.1 t C ha^–1^ at Langeweide, 1.6 t C ha^–1^ and 0.8 t C ha^–1^ at Hommerts C and H, respectively, 1.7 t C ha^–1^ at Lytse Deelen C and H and 2.6 t C ha^–1^ at De Burd C and T. Carbon application rates from slurry were derived using concentration value of 30 kg C t^–1^ and a density of 1 t m^–3^ (Hanegraaf et al. 2021).

### 2.2 Flux Measurements

EC systems adhered to a project standard configuration with only a couple of exceptions. The open‐path Licor LI‐7500DS was used to measure gas concentrations of CO_2_ and H_2_O and the open‐path Licor LI‐7700 was used to measure gas concentrations of CH_4_ (LI‐COR Biosciences, Lincoln, USA). At Langeweide a closed‐path Licor LI‐7200 was used instead of a LI‐7500DS. Gill Windmaster sonic anemometers (Gill Instruments, Hampshire, UK) measured three‐dimensional wind speed at all sites except Zegveld and Langeweide. At Zegveld a Metek uSonic‐3 Cage MP sonic anemometer (Metek GmbH, Elmshorn, Germany) was used and at Langeweide a Gill HS‐50 was used. Three‐dimensional wind speed and gas concentrations were recorded at a 10 Hz frequency. Raw data were recorded using Licor Smartflux 3 systems, which also uploaded the data to a central database via mobile internet connections. The height of the EC systems varied per location (Table 2). At some sites, the height of the instruments varied over time, either due to increases in canopy height or the desire to increase the flux footprint area. Additional information on flux processing is provided in Appendix A.

To exclude fluxes from unwanted areas, such as built‐up areas and or areas with vastly different land use at heterogeneous sites, wind direction and/or flux footprint filters were used. This is often an issue in the Netherlands where fields tend to be small. Where applied, the flux footprint filter method worked by calculating the footprint using the Kljun et al. (2015) two‐dimensional flux footprint prediction model for each timestep and then determining the weighted flux area within the target source area. The model was applied even when the displacement height was estimated to be within the roughness sublayer (e.g., during periods of high cattail) to gain a rough estimate of the flux source. The target source here is defined as the area of land with the desired land use and vegetation type. As an example, the target source area at Zegveld was the field that contained cattail vegetation and the unwanted area is the surrounding pasture, ditches and roads that may creep into the footprint depending on the wind speed and direction. A minimum threshold of 50% of the target area was used for all sites, and sites with stronger heterogeneity that would strongly bias measurements had a higher threshold (e.g., Zegveld) (Table 2). The mean footprint coverage in the target area after applying the minimum threshold is also presented in Table 2 to indicate how representative the measurements are, where the mean per cent coverage of the target area ranged from 62% to 99%. The target areas, mean footprint contours and locations of EC mast are provided for each site are shown with aerial imagery (Figures S1.1–S1.13). Timesteps where it was known or suspected that livestock emissions were measured, particularly at the pasture and pasture WIS sites, were excluded from the analysis. This was through the use of field logs and/or where the data showed uncharacteristically high emissions during daytime (grazing hours). After filtering for data quality and footprint considerations, the amount of data points remaining for analysis within the date range varied across the sites (Table 2). The mobile pasture sites had very few data points remaining, ranging between 1% and 2% of the total, whereas permanent masts had up to 33% of data points remaining. More comprehensive missing data statistics per site‐year are presented in Table S1.2. There was a nighttime bias in gaps, where the median percentage of data missing from nighttime for a site was 87% compared to the daytime of 80% across all sites. For sites that covered complete site‐years, the median missing nighttime percentage was 84% and daytime was 76%. This is unsurprising due to the poorly turbulent conditions that occur more often at nighttime. The percentage gaps belonging to each season in complete site‐years were nearly uniform, with slightly more gaps occurring in winter and autumn (median gaps, winter: 83%, spring: 78%, summer: 75%, autumn: 81%).

A standard meteorological station was co‐located nearby most EC systems. The station was not always within the same field, but sometimes located on an adjacent field or at least within the same property. The Duinigermeer lake EC site had no meteorological station and data from the Weerribben (~7.5 km northwest) was used. Because the biometeorological measurements normally occurred in a single plot or in a cluster close to the EC tower, they may not necessarily represent the full variation in conditions over the flux footprint but rather the land conditions (which dominate the footprints). The variables recorded included air temperature, relative humidity, atmospheric pressure, wind speed and direction, photosynthetic photon flux density (PPFD), soil temperature and water content, redox potential and groundwater levels. Instrument and logging information is provided in Appendix B.

### 2.3 Driver Analysis

Explanatory variables of FCH_4_ were visually explored to examine the principal drivers of fluxes at our study sites. The exploration of the drivers was grouped by land use classification to compare broad differences between the land use classes. We chose to present the analysis this way as it conveniently summarises the impact of land use, which is important in the context of peatland rewetting and land use change, but also due to the number of measurement sites in this study where site‐by‐site analysis would be more difficult to evaluate briefly. The explanatory variables inspected were soil (TS) or air (TA) temperature, groundwater level (GWL), redox potential (REDX), soil water content (SWC), gross primary production (GPP) and ecosystem respiration (R_eco_).

The importance of drivers was also assessed using Shapley values from machine learning models are described in the next section. Shapley values are derived using cooperative game theory, where the original concept was developed to estimate individual players importance in a collaborative team (Shapley 1953). Shapley values have since been adapted to explain the outputs of machine learning models and the approach is model agnostic (Lundberg and Lee 2017). The relationship between machine learning model features (explanatory drivers) and model output can be interpreted by assuming that the players in a game are the features where the prediction is the payout. The Shapley values essentially inform how to fairly distribute the payout to the features, that is, the contribution of a feature value to a prediction. Shapley values for an individual prediction are computed through permutation of all possible feature values and then by calculating the difference when each feature is included or removed, thereby measuring the impact of features on the prediction and their marginal contribution. The assessment of feature importance can be interpreted by the magnitude and sign of the Shapley values, where positive values indicate a positive contribution to the model prediction and vice versa, where negative values detract from the prediction. However, in this study, only mean absolute Shapley values are used in order to efficiently summarise the relative importance of drivers to model predictions. Shapley values analysis was conducted using the best‐performing machine learning model.

### 2.4 Gapfilling and Annual Budgets

Methane fluxes were gapfilled using the machine learning (ML) framework presented in Irvin et al. (2021). Four ML models were evaluated: lasso regression, artificial neural networks (ANN), random forest (RF) and gradient‐boosted decision trees (XGBoost). A brief description of the technical approach is provided in Appendix C. We used two sets of predictor variables for each site in our study. The first set is the so‐called ‘baseline’ set that has been used to gapfill FLUXNET‐CH4 data sets (Knox et al. 2019; Delwiche et al. 2021). The baseline set includes three temporal and four meteorological predictors. The temporal predictors are sine and cosine functions with a yearly wavelength and the decimal day of the year delta, and the meteorological variables are air temperature (TA), incoming shortwave radiation (SW_IN), atmospheric pressure (PA) and wind speed (WS). The second set, ‘all’, includes the variables in the baseline set and adds all available predictors with good temporal coverage. These extra variables include the additional meteorological predictors incoming photosynthetically active radiation (PPFD_IN), vapour pressure deficit (VPD), relative humidity (RH), precipitation (P), wind direction (WD, decomposed into *x* and *y* planes), soil temperature (TS) and soil water content with depth (SWC), redox potential with depth (REDX), groundwater level (GWL) and carbon and energy fluxes. The energy fluxes used were the sensible heat flux (H) and latent heat flux (LE) and the carbon fluxes used were the net ecosystem exchange (NEE), ecosystem respiration (R_eco_) and gross primary production (GPP). R_eco_ and GPP were partitioned from NEE using the nighttime partitioning approach (Reichstein et al. 2005). If there were missing predictor data, they were gapfilled. Biometeorological measurements were first gapfilled using the procedure described in Appendix B. Carbon and energy fluxes were first gapfilled using the marginal distribution sampling (MDS) algorithm (Reichstein et al. 2005). If gaps still remained (e.g., due to long gaps exceeding 60 days), they were imputed using year‐month‐hour means, followed by month‐hour means if gaps still remained. Mean imputation is standard in ML (Irvin et al. 2021).

The temporal data coverage of additional predictor variables was often poorer than the baseline set. This could occur when, for example, a sensor was installed much later than the flux tower. In addition, the additional predictors were not the same for all sites as some variables were site specific, or if a predictor variable could not be robustly gapfilled. As a result, the all‐predictor set often did not have the same temporal data coverage the baseline set, and the variables included for the all‐predictor set were site specific. We chose this approach as it maximised the amount of data that could be potentially gapfilled in the baseline set, while still providing an opportunity to see if the additional predictors in the all‐predictor set improved gapfilling performance and revealed insights into the importance of specific variables. The temporal coverage and predictor variables included in each site predictor‐set pair are presented in Table S3.1. The performance of the models was judged using the coefficient of determination (*R* ^2^), the mean absolute error normalised by the standard deviation (nMAE) and the mean bias.

We also gapfilled FCH_4_ using the MDS look‐up table approach which is widely used for gapfilling NEE but has also been used for FCH_4_ (Kim et al. 2020). The MDS approach is limited to only three predictor variables. We used five permutations of MDS predictor variables instead of seven as in Irvin et al. (2021) because we limited the combinations to only the four meteorological variables available in the baseline set. The MDS approach was evaluated using the same site training and testing data sets as in the ML approach.

Annual budgets were calculated as the cumulative sum of FCH_4_ from the ensemble means for each algorithm. The uncertainty of the models is the 95% confidence interval from the 10 models in the ensemble for the ML approach and five models in the MDS approach. In all approaches, gaps longer than 60 days were filled. There were a total of eight occurrences of gaps longer than 60 days which affected four sites (Table S1.3), however, from these only four site‐years are affected where we report annual totals. Of those four site‐years, none of those 60‐day gaps included summer. Moreover, ML methods have been shown to have better skill at filling longer gaps compared to MDS (Zhu et al. 2023), and MDS has been shown to be ineffective when gaps exceed 12 days for FCH_4_ (Moffat et al. 2007). Annual totals were not calculated for the mobile tower pasture sites in Table 2 due to the poor data coverage.

The machine learning framework of Irvin et al. (2021) is implemented in Python (Van Rossum and Drake 2009) using the scikit‐learn (Pedregosa et al. 2011) and xgboost (Chen and Guestrin 2016) packages. MDS gapfilling was performed using the REddyProc package (Wutzler et al. 2018). Shapley value analysis was conducted using the SHAP Python package (Lundberg and Lee 2017). Data and model outputs were analysed using R (v4.3.2) (R Core Team 2023) in combination with tidyverse packages (Wickham et al. 2019).

## 3 Results

### 3.1 FCH_4_ Observations

**FIGURE 2:**
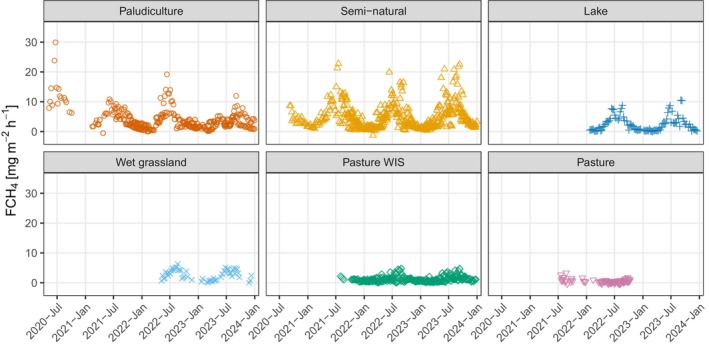
Timeseries of measured and filtered weekly median methane fluxes (FCH_4_). The points are coloured by their land use but represent the daily median for an individual measurement site.

**FIGURE 3:**
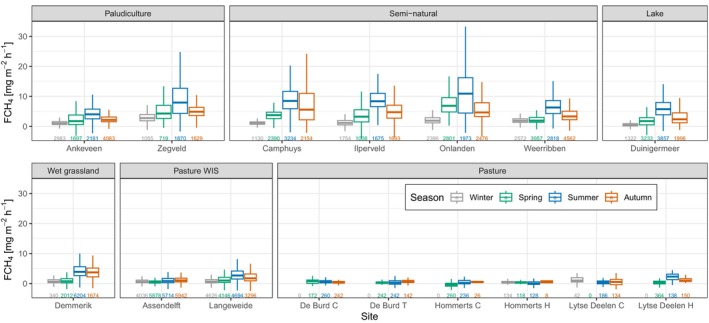
Boxplots of half‐hourly methane fluxes (FCH_4_) for each eddy covariance measurement site and grouped by land use. Seasons are northern hemisphere meteorological seasons. The numbers below each box show the number of data points (*n*) in each box. Values beyond the boxplot whiskers have not been plotted to enhance the view of the flux distributions but they are presented in Figure S2.1.

The timeseries of FCH_4_ measured by EC show clear seasonality of emissions with the highest emissions measured in the northern hemisphere summer in all land classes except for pasture (Figure 2). Sites within the semi‐natural sites and paludiculture land uses had the highest weekly median CH_4_ emissions compared to the other land uses, with emission rates often exceeding 10 mg CH_4_ m^−2^ h^−1^. FCH_4_ across all sites had a typical positively skewed distribution, where the mean and median were 3.82 mg CH_4_ m^−2^ h^−1^ and 2.55 mg CH_4_ m^−2^ h^−1^, respectively. The median flux of all paludiculture data was 2.63 mg CH_4_ m^−2^ h^−1^, however, there was a difference in median flux rates between the two paludiculture sites, Ankeveen and Zegveld, where Ankeveen had a median flux of 1.92 mg CH_4_ m^−2^ h^−1^, and Zegveld was 4.81 mg CH_4_ m^−2^ h^−1^. Median emission rates were similar for the semi‐natural sites Camphuys (4.83 mg CH_4_ m^−2^ h^−1^), Onlanden (4.80 mg CH_4_ m^−2^ h^−1^) and Ilperveld (4.09 mg CH_4_ m^−2^ h^−1^), but the median flux for the Weerribben was lower (2.78 mg CH_4_ m^−2^ h^−1^). The lake site Duinigermeer had a similar median flux rate to the Weerribben of 2.76 mg CH_4_ m^−2^ h^−1^. Demmerik had a median flux rate of 3.24 mg CH_4_ m^−2^ h^−1^. There was a difference between the two pasture WIS sites, where the median fluxes of Assendelft and Langeweide were 0.74 mg CH_4_ m^−2^ h^−1^ and 1.36 mg CH_4_ m^−2^ h^−1^, respectively. The median flux rate of all pasture sites was the lowest at 0.42 mg CH_4_ m^−2^ h^−1^ and site medians ranged from 0.01 mg CH_4_ m^−2^ h^−1^ to 0.77 mg CH_4_ m^−2^ h^−1^. Note that the summary statistics here have not been controlled for the uneven temporal sampling distribution across sites. The boxplots of site emissions also clearly display the seasonal nature of FCH_4_ (Figure 3).

Two of the sites with semi‐natural vegetation, Camphuys and particularly Weerribben, exhibited a strong diurnal pattern of higher emissions during daytime in summer and autumn (Figure S2.2). For example, at the Weerribben the largest difference between median hourly day and night emission rates during summer was around 5 mg CH_4_ m^−2^ h^−1^. The Zegveld paludiculture site also showed a similar diurnal pattern but it was weaker. The lake Duinigermeer showed the inverse and a pattern of higher emissions during summer nights. The remaining sites lacked clear differences in diurnal emissions.

### 3.2 Drivers

**FIGURE 4:**
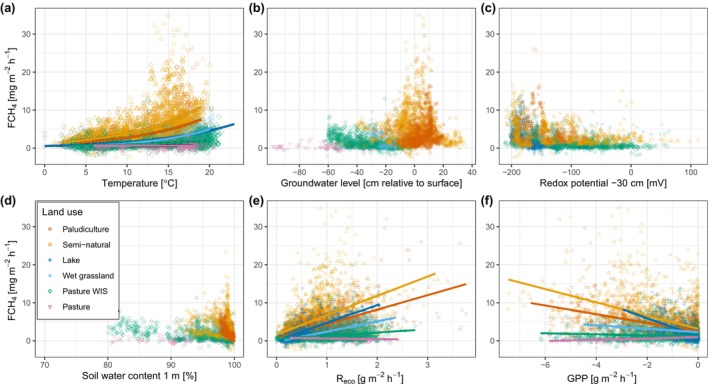
The relationship between daily median methane fluxes (FCH_4_) and (a) surface soil temperature (5, 10 or 15 cm; depending on site availability), except for lake where air temperature is used due to the lack of data (b) groundwater level relative to the surface, (c) redox potential at 30 cm below the surface, (d) soil water content in the top 1 m, (e) ecosystem respiration (R_eco_), and (f) gross primary production (GPP). Driver values corresponding to the daily median flux were selected for plotting. Non‐linear (plot a) and linear (plots e, f) regressions have been included to indicate trends where appropriate.

There was a positive relationship between temperature and daily median FCH_4_ for all land uses (Figure 4a). Paludiculture, natural vegetation, lake and wet grassland show the strongest exponential temperature responses. There is a pattern of increasing FCH_4_ with higher GWLs (Figure 4b). Across all land uses, there is a GWL breakpoint around –15 cm emissions start to steadily rise. Some emissions of FCH_4_ are lower at higher GWLs, but this is driven by covariation with other drivers (mainly temperature). Once the temperature is accounted for, there is an almost linear increase of FCH_4_ emission with GWL across all data (Figure S2.3). There is also a trend of high emissions in the pasture WIS land use with deeper GWLs, which will be discussed later in detail but it is most likely related to wide ditches in the Langeweide field site. There is a trend of higher FCH_4_ with lower values of redox potential at –30 cm (Figure 4c). The relationship with redox per measured depth interval is shown in Figure S2.4, where the same trend as in Figure 4c is shown at all depths except for pasture WIS at –10 cm. Spearman rank correlations of redox potential and FCH_4_ were mostly negative and stronger with depth (Table S2.1), except in some cases, where the upper soil zone can be expected to be aerobic (e.g., in pasture land uses). The plot of soil water content and FCH_4_ shows higher emissions occur when the soil profile is close to saturation (Figure 4d). In all land uses except for pasture, there is an overall positive relationship of higher daily R_eco_ and FCH_4_ as well as higher FCH_4_ with higher daily uptake of CO_2_ (GPP) (Figure 4e,f). There is a weak response of FCH_4_ to Reco and GPP in pastures with WIS, and the relationship increases in strength, in order, for wet grassland, paludiculture, lake and semi‐natural land uses.

### 3.3 Gapfilling

The performing MDS parameter combination that had the best test set performance on average on two out of the three performance metrics (*R* ^2^ and nMAE) was TA, WS and PA and that set is used for further comparisons (Figure S3.1). The median performance of MDS (*R* ^2^ = 0.56, nMAE = 0.42, Bias = –0.54 nmol m^–2^ s^–1^) was competitive to the ML methods with the baseline‐predictor set (Figure S3.2). The XGBoost (*R* ^2^ = 0.62, nMAE = 0.38, Bias = 0.20 nmol m^–2^ s^–1^) and RF (*R* ^2^ = 0.64, nMAE = 0.37, Bias = 0.66 nmol m^–2^ s^–1^) models performed the best out of the models with the baseline‐predictor set, where RF had a slightly higher *R* ^2^ and lower nMAE, but the mean bias was lower for XGBoost. Model performance generally improved across the three metrics for ML models using the all‐predictor set compared to the baseline (Figure S3.2) and XGBoost and RF had the highest performance again on average. For the all‐predictor set, XGBoost had median metrics of *R* ^2^ = 0.67, nMAE = 0.38, Bias = –0.82 nmol m^–2^ s^–1^ and RF had *R* ^2^ = 0.62, nMAE = 0.38, Bias = –0.35 nmol m^–2^ s^–1^, where XGBoost had a higher median *R* ^2^, the same nMAE, but a worse mean bias than RF. As a result of the similar baseline‐predictor set performance and slightly better all‐predictor set performance, XGBoost models were chosen for further analysis.

**FIGURE 5:**
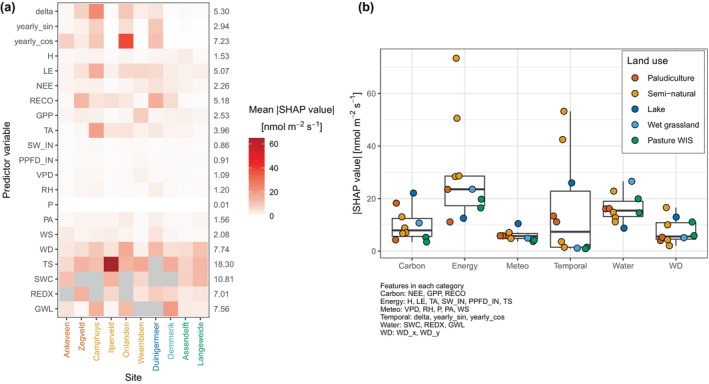
(a) Heatmap of mean absolute Shapley (SHAP) values for XGBoost models using the all‐predictor set. Boxes are filled with grey if the variable was not included for model training at a site. If there were multiple probes or probes with depth, the SHAP values were grouped and summed. The right *y*‐axis shows the average mean asbolute SHAP value for the input feature across all sites. Individual variable values are presented in Figure S3.3. (b) Summary of SHAP values classified into six main overarching categories of carbon, energy, meteorology (meteo), temporal, water and wind direction (WD). Features were classified into six main categories and their mean absolute SHAP values per feature per site were summed. The features included in each category are shown in the plot footnote.

The mean absolute Shapley values of the XGBoost models with the all‐predictor set are presented in Figure 5a to investigate explanatory drivers. Shapley values were calculated for the 10 XGBoost models using their corresponding training data sets. The mean absolute values for each model were calculated and then a final mean was calculated by averaging the mean absolute values. A convenient feature of Shapley values is that they are additive, and multiple‐related input features, such as soil temperature with depth or multiple water‐level probes, have been summed to simplify the plot. The most important features on average to model output are soil temperature (TS: 18.30 nmol m^–2^ s^–1^), soil water content (SWC: 10.81 nmol m^–2^ s^–1^), wind direction (WD: 7.74 nmol m^–2^ s^–1^), groundwater level (GWL: 7.56 nmol m^–2^ s^–1^), yearly cosine (7.23 nmol m^–2^ s^–1^), redox potential (REDX: 7.01 nmol m^–2^ s^–1^) and day of year delta (5.30 nmol m^–2^ s^–1^). Across the sites, soil temperature was ranked the most important driver in five sites, in the top three features for three sites, the fifth for one site, and for one site (Duinigermeer) it was not included. Some features that were not universally important but were at some sites include GPP at Weerribben, R_eco_ at Zegveld and Duinigermeer and latent heat flux at Camphuys and Weerribben. Land uses with multiple sites did not consistently have the same features as important, however, there was some similarity for pasture WIS, where the top three features were the same for both Assendelft and Langeweide (TS, SWC and WD). Some other patterns that are observable are that the energy and carbon fluxes were usually important to the semi‐natural and lake sites, whereas the temporal predictors were found to not be useful at the wet grassland and pasture WIS sites.

Since there are several input features included in the ML training that correlate with each other, we classified the variables into categories to investigate which overarching drivers are important. The input features were classified into the categories of carbon (NEE, R_eco_ and GPP), energy (TA, TS, SW_IN, PPFD_IN, H and LE), meteorology (VPD, RH, P, PA and WS), temporal (delta, yearly sine and cosine), water (GWL, SWC and REDX) and wind direction (WD), and the mean absolute Shapley values were summed per site and the results are shown in Figure 5b. Using the median of the groups, energy (23.5 nmol m^–2^ s^–1^) was the dominant factor affecting model output, followed by water (15.4 nmol m^–2^ s^–1^), carbon (7.9 nmol m^–2^ s^–1^), temporal (7.4 nmol m^–2^ s^–1^), meteorology (5.7 nmol m^–2^ s^–1^) and wind direction (5.6 nmol m^–2^ s^–1^).

### 3.4 Annual Totals

**FIGURE 6:**
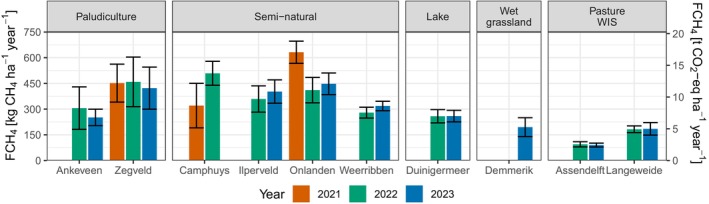
Annual totals of methane fluxes (FCH_4_) gapfilled with XGBoost machine learning models. The uncertainty bars represent the 95% confidence interval derived from the variance of the 10 gapfilled timeseries. Annual totals for a site‐year may be from the baseline or all‐predictor set, whichever had the lowest uncertainty. The annual totals are also presented in Table S3.2. The right‐hand y‐axis displays the annual totals in CO_2_ equivalents using the 100‐year non‐fossil global warming potential of methane (27.0).

Annual totals of FCH_4_ were selected from the baseline and all gapfilled sets were based on availability and annual uncertainty. If annual estimates from both a baseline and all‐predictor set were available for a budget year, the annual total with the lowest uncertainty was chosen. Annual totals of FCH_4_ (total ± 95% CI) ranged from 90 ± 11 to 632 ± 65 kg CH_4_ ha^–1^ year^–1^ (Figure 6 and Table S3.2). The annual totals were on average highest from sites with natural vegetation and ranged from 279 ± 32 to 632 ± 65 kg CH_4_ ha^–1^ year^–1^. The annual emission of the lake was slightly below the range of natural vegetation for the two budget years (258 ± 39 kg CH_4_ ha^–1^ year^–1^ and 259 ± 34 kg CH_4_ ha^–1^ year^–1^). Paludiculture annual totals ranged from 251 ± 48 to 459 ± 145 kg CH_4_ ha^–1^ year^–1^, however, there was a large difference between the two sites, Zegveld and Ankeveen, where Zegveld emitted 153 and 171 kg CH_4_ ha^–1^ year^–1^ more CH_4_ annually for 2022 and 2023 than Ankeveen, respectively. Differences between these two sites can be mostly expected due to differences in site set‐ups, including vegetation composition and water quality, which are discussed later. Wet grassland only had one annual estimate of 195 ± 55 kg CH_4_ ha^–1^ year^–1^ for the year 2023. Emissions from pasture WIS sites ranged from 90 ± 11 to 185 ± 37 kg CH_4_ ha^–1^ year^–1^ and emissions between the two sites Assendelft and Langeweide also differed markedly, again which can be mostly expected due to differences in open water footprint coverage (Table 2). The annual totals from the other models (MDS and ML) are provided in Table S3.3 for reference.

Two sites, Camphuys and Onlanden, showed higher interannual variability in 2 years. There were large changes in GWL management at both sites between years that showed variability. Camphuys had a mean annual and (summer) GWL of −6 cm (−20 cm) and 3 cm (1 cm) in 2021 and 2022, respectively. The annual emissions in 2021 and 2022 at Camphuys were 320 ± 130 kg CH_4_ ha^–1^ year^–1^ and 509 ± 70 kg CH_4_ ha^–1^ year^–1^. Similarly, at Onlanden emissions in 2021 were higher than in 2022 and 2023. Mean annual and (summer) GWL at Onlanden were 10 cm (3 cm), 4 cm (–6 cm) and 10 cm (–5 cm) for 2021, 2022 and 2023, respectively, and the corresponding annual totals for these years was 632 ± 65 kg CH_4_ ha^–1^ year^–1^, 411 ± 74 kg CH_4_ ha^–1^ year^–1^ and 448 ± 63 kg CH_4_ ha^–1^ year^–1^, respectively. In comparison, Ilperveld and Weerribben, which had little change in GWL management between the 2 years, also only had small differences in their annual totals.

**FIGURE 7:**
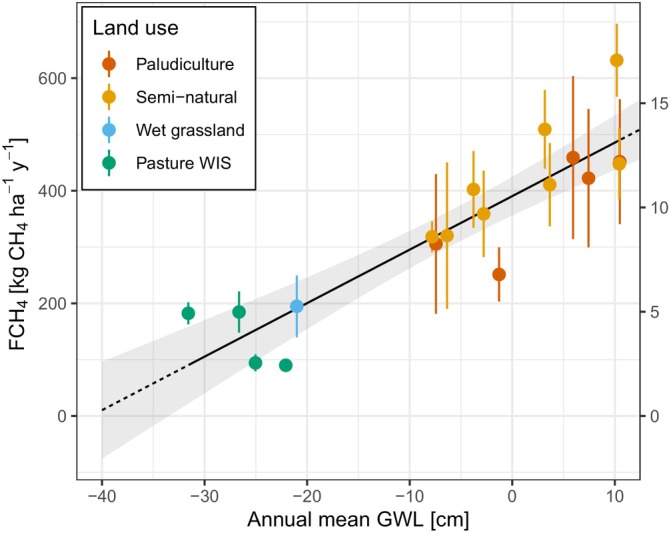
The relationship between mean annual groundwater level (GWL) and annual methane fluxes (FCH_4_). The black line is a linear model that was fit to the data (FCH_4_ = 390.5 + 9.5GWL; Pearson's *R* ^2^ = 0.80, *p* < 0.001, *n* = 18) which becomes dashed outside the observed GWL range. The shading of the line indicates the 95% confidence interval of the regression fit. The y‐axis error bars for each point show the 95% confidence interval of annual FCH_4_.

The relationship between the mean annual GWL and annual FCH_4_ for our study sites is shown in Figure 7. Our data have a positive linear trend where a higher GWL is associated with higher annual FCH_4_. A linear model was fit to the data and the relationship is FCH_4_ = 390.5 + 9.5GWL (Pearson's *R* ^2^ = 0.80, *p* < 0.001; *n* =18; RMSE = 64 kg CH_4_ ha^–1^ year^–1^). The relationship between annual totals and mean summer GWL was also tested, however, the fit was worse (FCH_4_ = 432.13 + 6.7GWL, Pearson's *R* ^2^ = 0.69; *p* < 0.001; *n* =8; RMSE = 80 kg CH_4_ ha^–1^ year^–1^). A non‐linear logistic regression was also attempted with the summer GWL data with only a small improvement in the fit (*R* ^2^ = 0.74; RMSE = 74 kg CH_4_ ha^–1^ year^–1^).

## 4 Discussion

### 4.1 Understanding Emissions

Temperature of the soil or air was strong predictors of CH_4_ fluxes and was positively associated. Soil temperature, in particular, has been widely found to be one of the controls of emissions (e.g., Delwiche et al. 2021; Kroon et al. 2010; Schrier‐Uijl et al. 2010a; Morin et al. 2014; Turetsky et al. 2014; Wilson et al. 2016; Peacock et al. 2021), given that it is one the main drivers that stimulates methanogenic archaea (Segers 1998; Christensen et al. 2003). It was also found to be the most important predictor of emissions when models were trained to the FLUXNET‐CH4 data set (Irvin et al. 2021) and also in this study with the highest mean absolute Shapley values (Figure 5). Other studies have reported the relationship with temperature can be seasonally dependent (Koebsch et al. 2015; Chang et al. 2021) since it is also driven by other factors such as water inundation and substrate availability.

Our data showed a GWL window between –10 and 10 cm, where emissions of FCH_4_ were the highest (Figure 4b). A similar window of high emissions was found for rich fens in a northern peatland data set by Turetsky et al. (2014) and by Levy et al. (2012) who measured CH_4_ emissions from United Kingdom soils. Some emissions were lower with higher GWLs or vice versa, however, this is most likely due to the co‐dependency of other drivers, as demonstrated by the near‐linear increase of FCH_4_ with GWL once the temperature is removed (Figure S2.3). In some cases, lower GWL was associated with high CH_4_ emissions in non‐pasture land uses. Event‐based carbon imports by irrigation water could explain these patterns (Mander et al. 2014). The relationship between GWL and CH_4_ emissions was not straightforward per land use class, as shown in Figure 4. Since the GWLs are bound to land use and management, their range is mostly limited. Prior work has shown that GWL is an important or limiting factor when the range is large and whether is crosses depth thresholds for methane emission (i.e., surface fluctuation) (Knox et al. 2019, 2021; Cui et al. 2024). Clear patterns were not observed for paludiculture and semi‐natural land uses where the range in GWLs is mostly limited.

CH_4_ emissions from the pasture WIS class appeared to increase with deeper GWLs (Figure 4b). The observations with this trend come from the Langeweide site which has a relatively high proportion of ditches in the flux footprint (18% open water, Table 1). Ditch and border ditch zones in agricultural meadows are known to be strong sources of methane (Schrier‐Uijl et al. 2010b). The site is also grazed by cattle, and despite best efforts to remove the impacts of cattle by excluding periods with grazing, they may still be present. The Assendelft site, which had a small percentage of open water in the footprint in the form of drainage ditches, also had correspondingly lower fluxes of CH_4_. Direct observations of terrestrial and ditch emissions using chambers would be useful for confirmation and these measurements are currently being conducted. Previous measurements of ditch emissions in the Netherlands have shown emission rates range between 1.2 and 39.3 mg CH_4_ m^–2^ h^–1^ (Schrier‐Uijl et al. 2011) and a separate study estimated a mean annual emission factor of 2144 kg CH_4_ ha^–1^ year^–1^ (Hendriks et al. 2024), which shows that it is plausible that the emissions in Langeweide are mostly of ditch or ditch border zone origin but further assessment is required. More broadly, the role of open water emissions across all sites needs to be examined in more detail since they can be hotspots of CH_4_ emissions, where Franz et al. (2016) found open water to have fourfold higher emissions than emergent vegetation.

All sites, except Weerribben, have undergone relatively recent changes in groundwater level management, ranging from very recent (Camphuys and Ankeveen in 2019) to medium to decadal term changes (e.g., Zegveld 2016; Ilperveld and Onlanden in 2012; Demmerik 1980s; Assendelft 2018). In this study, unchanged control plots where methane fluxes were measured are not available. It is therefore difficult to quantify the change of the FCH_4_ because of re‐wetting and conversion to paludiculture or natural wetland. Moreover, the short time between the start of measurements and conversion may include effects from the adjustment of vegetation and soil carbon stocks to the new wetter conditions, which hold the risk of elevated FCH_4_ emissions (Hahn‐Schöfl et al. 2011; Harpenslager et al. 2015). It cannot be excluded that this has affected some of the sites, in particular the sites with the most recent GWL increase. Abrupt GWL changes exceeding the range of previous GWL may result in temporary increased availability of labile organic substrate for methanogens, from soil carbon reservoirs such as roots, manure, and remains of vegetation that is not adapted to higher GWLs (Franz et al. 2016; Hemes et al. 2018). Based on the uncertainty introduced by the temporary effects of GWL change, the CH_4_ emission data from semi‐natural sites in this paper may not be representative of long‐term emission rates for wetland nature.

Diurnal patterns are known to be particularly dynamic and can depend upon biotic and abiotic controls (Bansal, Tangen, and Finocchiaro 2018). We found a clear diurnal cycle in sites dominated by reed and cattail vegetation, where higher emissions were observed during the day. Reeds and cattails are known to have more prominent diel emissions due to their internal gas transport mechanisms which are five times more efficient than diffusion (Brix, Sorrell, and Orr 1992; Brix, Sorrell, and Lorenzen 2001). The diurnal cycle in reed vegetation has been observed in another study with EC by van den Berg et al. (2016), who also found that the effect was only present when there was living green vegetation present. This effect was not explicitly tested here, but it was visible with the seasonal differences in diurnal cycles in Figure S2.2. The diurnal effect was strongest at the Weerribben site in our study, where the lower emissions during nighttime can be partially explained by the presence of *Sphagnum* which is associated with microbial oxidation of CH_4_ (Kip et al. 2010), leaving the gas transport by reeds as the dominant source of terrestrial emissions. The Duinigermeer had higher emissions during the night than the day, which is the inverse of what Schrier‐Uijl et al. (2011) found for Dutch ditches and lakes. However, Schrier‐Uijl et al. (2011) remarked that diurnal variations in fluxes could arise because oxygen production is greater during the day and that can stimulate CH_4_ oxidation. The lake in our study had abundant plant activity during the warmer months and the oxygen produced from photosynthesis may have enhanced the oxidation of CH_4_ during the day (e.g., van Grinsven et al. 2021). Higher nighttime emissions may also be because of convective mixing that brings CH_4_‐rich water to the surface from the bottom of the lake, as well as stimulating bubble release from the lake sediment (Podgrajsek, Sahlée, and Rutgersson 2014).

The effect on CH_4_ emission of nutrients and carbon import by water and fertilisation has not been evaluated in detail yet. Paludiculture sites receive irrigation water that contains nutrients and particulate or dissolved carbon, while sites that have a floodwater storage function (Onlanden) receive nutrients and carbon by flooding. Nutrients interact with CH_4_ emission by increasing vegetation primary production, determining vegetation composition, and by interacting with the microbial community, affecting methanogenesis and methanotrophy. Such data are not readily available for all sites in this study, and where available this analysis needs to be conducted on the site scale rather than the broad scale approach in this study. Similarly, the effect of water chemistry and salinity needs to be further investigated, where the availability of different terminal electron acceptors (e.g., ferric iron, nitrate, sulphate) influences the rate of CH_4_ oxidation (Gupta et al. 2013) and where our redox measurements may be useful (e.g., Boonman et al. (2024)). Further, the role of emission hotspots has not been evaluated across all sites.

### 4.2 Gapfilling Drivers

The Shapley value analysis showed that many input features contribute to model predictions and reinforce the multi‐driver dependency of methane fluxes as found by other studies (Kim et al. 2020; Irvin et al. 2021). Soil temperature was found to be the most important feature in the XGBoost models, a result that was also found by Irvin et al. (2021) for RF feature importance for FLUXNET‐CH_4_ sites. The fuzzy temporal predictors (delta, yearly sine and cosine) were often useful features and captured the highly seasonal nature of the FCH_4_. Interestingly, the temporal predictors were not useful in the wet grassland and pasture WIS sites which may be because they are more impacted by land management (e.g., vegetation harvesting, grazing). The inclusion of additional predictors that represent changes in vegetation, such as the normalised difference vegetation index or leaf area index, may be beneficial for grasslands, but also for other land uses since vegetation dynamics have been shown to be important for CH_4_ transport (Vroom et al. 2022) and prediction (Räsänen et al. 2021). Assigning the input features into groups attempted to summarise which categories were the most important and to understand whether the theoretical primary controls on FCH_4_ were grasped by the ML models (Figure 5b). We found the theoretical controls were well represented, with energy (enzyme kinetics), water (anaerobic conditions) and carbon (substrate availability) were categories with the highest median importance. The high importance of wind direction indicated that there may be emission hotspots at our sites, but it is also a reflection of the heterogeneity of our study sites. More broadly, spatial variability of FCH_4_ is a common feature as often shown by point scale chamber measurements (Hendriks, van Huissteden, and Dolman 2010).

In this study, we only used the mean absolute Shapley values to efficiently gain a high‐level overview of the importance of features. However, the sign of the Shapley value is also informative to understand whether a feature is contributing to or from CH_4_ emission. Moreover, the magnitude and sign of Shapley values vary for individual predictions, and insight into temporal variability and interaction of feature behaviour on predictions may also be gained by analysing the individual values. Future work should examine the sign and magnitude to gain more insight into the role of input features and their effect on predictions and therefore insight into the drivers of CH_4_ emission.

### 4.3 Gapfilling Performance

The median variance explained by the XGBoost machine learning models for the test data was 62% and 66% for the baseline and all‐predictor sets, respectively. This indicates that only a few variables are required to achieve reasonable predictions. Interestingly, while the baseline‐predictor set performed relatively well compared to the all‐predictor set, the meteorological drivers included in the baseline set (TA, SW_IN, PA, WS) did not have high absolute Shapley values in the all‐predictor set (Figure 5). These variables would have strong correlations with other predictors in the all‐predictor set, such as air temperature with soil temperature and shortwave radiation with GPP. The amount of variance not explained in this study indicates that the models may, for example, be missing important features and/or require more data to learn and predict emission behaviour.

The performance of the XGBoost models in this study was lower than but comparable to the median performance for the test set for the models RF (*R* ^2^ = 0.79; nMAE = 0.27; Bias = 0.24 nmol m^–2^ s^–1^) and ANN (*R* ^2^ = 0.73; nMAE = 0.30; Bias = 0.18 nmol m^–2^ s^–1^) in Irvin et al. (2021), where RF and ANN were chosen as they were the better performing models. Differences in performance metrics could arise from the land use of the sites being gapfilled, the amount of data available per site and differences in predictors that were included during training. In another study of gapfilling strategies for CH_4_ fluxes, Kim et al. (2020) found RF to outperform two other ML algorithms (ANN and support vector machines (SVM)). While there are questions about the suitability of the MDS approach to gapfill FCH_4_ due to the limited number of drivers it can incorporate (Kim et al. 2020), we found that it could achieve reasonable performance compared to the machine learning approaches and annual totals derived using this method would likely be comparable, as Irvin et al. (2021) also found.

The 95% confidence interval of the presented annual totals, calculated from the variance of the cumulative sums of the 10 gapfilled timeseries, ranged from 11 to 145 kg CH_4_ ha^–1^ year^–1^ and a median uncertainty of 63 kg CH_4_ ha^–1^ year^–1^. In terms of percentage of the annual flux, the range was 9%–41% with a median of 18%. There was a clear Pearson's correlation (*r* = –0.59; *p* < 0.01) between the number of annual data points and the per cent annual uncertainty, indicating that data availability was clearly an issue. Many sites had low data availability due to need to exclude flux timesteps due to small parcel sizes and/or heterogeneous footprints, in addition to loss of timesteps due to maintaining data quality (e.g., well‐developed turbulence, high signal strength of the open‐path gas analysers). Moreover, some sources of uncertainty were not propagated in this study, such as the u* threshold. Irvin et al. (2021) showed that through extrapolating fluxes of high u* conditions to low u*, the error, while small, was not negligible.

### 4.4 Annual Totals

The semi‐natural vegetation sites had the highest average emissions of the land uses included in this study. These semi‐natural sites were nutrient rich with eutrophic vegetation, except for the Weerribben which is mesotrophic to eutrophic. The annual totals found for the Weerribben and Camphuys are comparable to previous studies dominated by reed vegetation, where Kankaala, Ojala, and Käki (2004) reported a range of 207–485 kg CH_4_ ha^–1^ year^–1^ in Finland and van den Berg et al. (2016) reported 311 kg CH_4_ ha^–1^ year^–1^ in Germany. The methane emissions of rewetted peatlands that are vegetated by *Typha* species can vary quite substantially, for example, 40–1700 kg CH_4_ ha^–1^ year^–1^ as compiled by Franz et al. (2016, table 5). Onlanden, which is dominated by *Typha*, is between the first quartile and the median of ranges of studies in Franz et al. (2016) (340–695 kg CH_4_ ha^–1^ year^–1^). The annual totals of the recently rewetted sites, Camphuys, Onlanden and Ilperveld, are also comparable to the annual FCH_4_ of the Horstermeer of 444 kg CH_4_ ha^–1^ year^–1^, another recently rewetted site in the Netherlands. The emissions of our sites were higher than the range of annual totals of natural fens in Irvin et al. (2021) that are in Finland, Sweden and the United States (84–208 kg CH_4_ ha^–1^ year^–1^), but they were in the range of marsh systems all located within the United States (435–1756 kg CH_4_ ha^–1^ year^–1^).

The mean annual emissions of lake Duinigermeer was 259 ± 26 kg CH_4_ ha^–1^ year^–1^. There are few studies reporting annual emissions of CH_4_ from freshwater lakes. Our annual totals are similar to those of a Swedish freshwater lake of 245–295 kg CH_4_ ha^–1^ year^–1^ reported by Jackowicz‐Korczyński et al. (2010). A wide range of emissions was found in a German lake that was a formerly drained fen between 120 and 537 kg CH_4_ ha^–1^ year^–1^ (Franz et al. 2016; Kalhori et al. 2024), however, the highest emissions were measured recently after rewetting and there is a strong decreasing trend over time. While freshwater lakes are a large global source of CH_4_ (Johnson et al. 2022), emissions can vary substantially across lakes based on several factors including lake area, depth, organic matter input and decomposition (Bastviken et al. 2004) and therefore comparisons are difficult and emissions may span a wide range. In addition, the emissions of our site could be interpreted as wetland emissions considering the presence of floating vegetation and reeds and cattail on the lake edges (Aubinet, Papale, and Vesala 2012), and indeed, the emission rates are comparable to those of the nearby terrestrial site Weerribben.

There are very few studies that report an annual total of CH_4_ for paludiculture, specifically for cattail paludiculture, and therefore, our results make an important contribution to quantify cattail paludiculture emissions. The annual paludiculture emissions of Zegveld were reported in another study that used a simple empirical temperature and water‐level model coupled in a Bayesian inference framework that tried to account for the mixed footprint due to the small parcel size. The annual emissions reported in that study ranged from 466 to 626 kg CH_4_ ha^–1^ year^–1^ (Buzacott et al. 2024), which are above the annual totals reported here but are within the 95% uncertainty bounds. Another study of cattail species in the Netherlands used a combination of chamber measurements and modelling, annual totals of FCH_4_ for *T. angustifolia* and *T. latifolia* of 369 kg CH_4_ ha^–1^ year^–1^ and 848 kg CH_4_ ha^–1^ year^–1^ were reported (van den Berg et al. 2024). Comparisons can also be made to natural or semi‐natural areas that have cattail species, where the annual emissions were similar to Onlanden in our study and in, for example, Günther et al. (2015), Franz et al. (2016), Petrescu et al. (2015), Rey‐Sanchez et al. (2018). While the annual totals are similar, there are likely to be several differentiating factors that may influence emissions, such as harvesting, aquatic carbon imports, and the recent land conversion including topsoil removal and abrupt raising of the GWL (Kreyling et al. 2021). It should be noted that there are other paludicrops other than cattail species, such as reeds, canary grass, *Azolla*, alder and *Sphagnum*, and the CH_4_ emissions can vary substantially (Beyer and Höper 2015; Huth et al. 2018; Kandel et al. 2020; Tanneberger et al. 2022; van den Berg et al. 2024).

There was a large difference in annual totals for our two paludiculture sites Zegveld and Ankeveen. Zegveld only has *T. latifolia* within the target measurement area, whereas Ankeveen has plots of different cattail species, sedges, rushes and reeds. The different vegetation species, and water levels in the subplots, would have different impacts on emission rates. We have measured vegetation‐dependent FCH_4_ emission rates using chamber systems at Ankeveen, however, it is difficult to assess the impact of particular plant species on the EC annual totals without a careful analysis of changes in emissions with coverage of the flux footprint of different species and it is beyond the scope of this study. Besides the differences in vegetation composition, differences in water quality and the amount of dissolved organic carbon in irrigation water between the sites may be at least partially responsible and they should be examined in future research.

The pasture WIS and wet grassland systems had annual totals ranging from 90 ± 11 to 185 ± 37 kg CH_4_ ha^–1^ year^–1^ and 195 ± 55 kg CH_4_ ha^–1^ year^–1^, respectively. Across site years, the emissions here are comparable to a previous study on Dutch meadows of 155–172 kg CH_4_ ha^–1^ year^–1^ (Schrier‐Uijl et al. 2014), German fen peat emissions of 68 ± 121 kg CH_4_ ha^–1^ year^–1^ (mean ± 1 SD) reported by Tiemeyer et al. (2016) and also the IPCC emission factor to shallow drained nutrient‐rich fens of 106 ± 139 kg CH_4_ ha^–1^ year^–1^ (mean ± 95% CI) (IPCC 2014). For comparison, nutrient‐rich deep‐drained grasslands have a default IPCC annual emission factor of 16 kg CH_4_ ha^–1^ year^–1^ (IPCC 2014) and deep‐drained German meadows have an annual emission of 11.2 kg CH_4_ ha^–1^ year^–1^ with a 95% CI of 0.6–86.4 kg CH_4_ ha^–1^ year^–1^.

### 4.5 Overriding Controls on Annual Totals

We found a strong relationship between the mean annual GWL and mean annual CH_4_ flux. Fundamentally, the overriding control is the land use because the land use also determines possible GWLs in areas where the GWL is closely managed, as is the case in the Netherlands. Interannual weather differences such as temperature also play an important role but variation in annual temperature between sites in the Netherlands is small and all sites have a similar mean annual temperature for a given year. An important note is that while we found a clear positive overall relationship between GWL and FCH_4_, within land uses there was not always a positive, as shown by pasture WIS (Figure 7). Here, it is likely that site‐to‐site differences play a large role, such as heterogeneity of the measurement area including vegetation, open water coverage, and spatial variability of water levels, amongst other factors. A reason for the poorer fit of the mean summer GWL may be that there is a bias towards drier conditions, where CH_4_ is more likely to be emitted during prolonged periods of higher GWL which may be captured better by the mean annual GWL. Alternatively, it may be another indication of emissions of CH_4_ from non‐terrestrial areas that the GWL‐FCH_4_ relationship does not capture.

**FIGURE 8:**
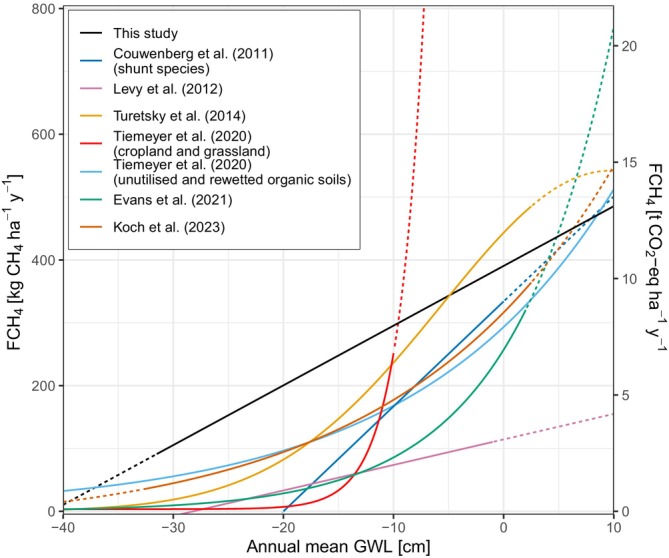
Comparison of relationships of annual mean groundwater level (GWL) and methane flux (FCH_4_) from continental Europe (Couwenberg et al. 2011), British sites (Levy et al. 2012), rich fens from North America and Nordic region (Turetsky et al. 2014), German sites for two categories (Tiemeyer et al. 2020), British and Irish sites (Evans et al. 2021) and Danish sites (Koch et al. 2023). Note that the Couwenberg et al. (2011) fit only applies to plant‐mediated transport (shunt) species. Lines become dashed outside the data or plotting ranges used for each study.

The relationship between mean annual GWL and annual FCH_4_ has been investigated by studies in continental Europe (Couwenberg et al. 2011), Britain (Levy et al. 2012), North America and Nordic peatlands (Turetsky et al. 2014), Germany (Tiemeyer et al. 2020), Britain and Ireland (Evans et al. 2021) and in Denmark (Koch et al. 2023) and each have found it to explain a high proportion of the annual variance of FCH_4_. In keeping with these studies, we also have shown the relationship with our data and have compared the fits with the aforementioned studies in Figure 8. Levy et al. (2012) is clearly lower than the other fits which is likely due to the high amount of bogs in their study which have lower CH_4_ emissions than fens. Our fit indicates higher annual FCH_4_ emissions with deeper GWL, however, the fits are closer once the GWL approaches the surface. Another reason for the difference is the measurement method, whereas the other studies except for Evans et al. (2021) mostly used chamber measurements for CH_4_ which would limit the influence of drainage ditches and open water areas which are high sources of CH_4_ (Peacock et al. 2021; Hendriks et al. 2024), where open water/ditches make up 16% and 24% of the mean footprint at Langeweide and Demmerik, respectively (Table 2). Constraining the emissions to only terrestrial sources is a difficult task with EC (e.g., Buzacott et al. 2024), particularly at our measurement sites which can be high heterogeneity and/or have comprehensive networks of ditches.

### 4.6 Future Research

This study presents the first large‐scale data collection and analysis of CH_4_ emissions from semi‐natural and agriculturally used peatlands in the Netherlands. Data collection at this scale will contribute to improve emission reporting of wetland and agricultural CH_4_ emissions in the Netherlands, for which the amount of data is hitherto very limited; wetland emissions are not reported due to a lack of data (Ruyssenaars et al. 2021). In this study, we limited ourselves to the peatlands on the coastal plain and we do not have sites in wetlands and peatland rewetting areas that occur on the higher Pleistocene sandy areas in the Netherlands. These areas contain remnants of former large areas of ombrotrophic peatlands and eutrophic and mesotrophic peatlands in river valleys (Verhoeven 2013) where different amounts in CH_4_ emissions can be expected.

A full assessment of emission and radiative forcing trade‐offs of different greenhouse gases for different land uses was out of scope of this study. Other than the fluxes of CO_2_, CH_4_ and where applicable N_2_O, close attention needs to be given to carbon imports, exports and long‐term storage potential which can decide whether an ecosystem or land use has a long‐term reduction in radiative forcing (Günther et al. 2020). This will be examined in future work. A longer timeseries covering a wider range of weather conditions and time since rewetting may also be required to judge this effectively. However, it is clear from the results that high rates of annual CO_2_ uptake and long‐term storage are required to fully offset emissions—in the range of at least 10–15 t CO_2_‐eq ha^–1^ year^–1^ for paludiculture and semi‐natural land uses when using the 100‐year global warming potential of 27.0 for non‐fossil CH_4_ (IPCC 2021). However, despite the increase in CH_4_ emissions, there is likely to be a lower net warming impact compared to a scenario where there is continued drainage of the peatlands (Günther et al. 2020).

## 5 Conclusions

This study presented a large‐scale collection of CH_4_ emissions measured at 16 sites across six land uses on Dutch peatlands along the coastal plain. Our data reinforce the typical controls found for CH_4_ emission, the most important of which were soil temperature and groundwater level, but there were strong interactions with different drivers across different land use and vegetation combinations. There was a clear average increase in CH_4_ emissions with increasing water levels and the highest emissions were observed with water levels above 10 cm below the surface. We found the tree‐based ML models, XGBoost and random forest, to provide the best gapfilling performance of FCH_4_. As with other studies, soil temperature was found to be the single most important predictor of FCH_4_ and we also observed a strong multi‐driver dependency of FCH_4_, as shown by the Shapley values. The gapfilling of the data resulted in 21 site‐years of annual flux totals. However, the uncertainty of annual totals was large for some site‐years where the low observations of FCH_4_ hindered performance. Semi‐natural land uses had the highest annual totals of FCH_4_ and ranged from 279 ± 32 to 632 ± 65 kg CH_4_ ha^–1^ year^–1^, followed by paludiculture 251 ± 48 to 459 ± 145 kg CH_4_ ha^–1^ year^–1^, lake 258 ± 36 to 259 ± 34 kg CH_4_ ha^–1^ year^–1^, wet grassland 195 ± 55 kg CH_4_ ha^–1^ year^–1^ and pasture WIS between 90 ± 11 and 185 ± 37 kg CH_4_ ha^–1^ year^–1^. We also found that there was a strong relationship between mean annual groundwater level and annual emission of FCH_4_ across all land uses, however, within land uses, this relationship did not always hold. Future work needs to examine the radiative forcing trade‐offs of different greenhouse gases across the different land uses and rewetting strategies to determine the net impact on the climate.

## Author Contributions

**Alexander J. V. Buzacott:** conceptualization, data curation, formal analysis, investigation, methodology, visualization, writing – original draft, writing – review and editing. **Bart Kruijt:** conceptualization, methodology, project administration, writing – review and editing. **Laurent Bataille:** data curation, writing – review and editing. **Quint van Giersbergen:** data curation, formal analysis, writing – review and editing. **Tom S. Heuts:** data curation, writing – review and editing. **Christian Fritz:** conceptualization, methodology, writing – review and editing. **Reinder Nouta:** data curation. **Gilles Erkens:** funding acquisition, project administration, writing – review and editing. **Jim Boonman:** data curation, formal analysis, writing – review and editing. **Merit van den Berg:** conceptualization, project administration, writing – review and editing. **Jacobus van Huissteden:** conceptualization, formal analysis, investigation, writing – original draft, writing – review and editing. **Ype van der Velde:** project administration, supervision, writing – review and editing.

## Conflicts of Interest

The authors declare no conflicts of interest.

## Supporting information


**Data S1.** Supporting Information.

**Data S2.** Supporting Information.

**Data S3.** Supporting Information.

## Data Availability

The data that support the findings of this study are openly available in Dryrad at https://doi.org/10.5061/dryad.1rn8pk140. The machine learning model scripts and output that supports the findings of this study are openly available in Zenodo at https://doi.org/10.5281/zenodo.14045079.

## Appendix A Flux Processing

### A.1

Turbulent fluxes were calculated using the covariance of vertical wind speed and respective scalars of interest using EddyPro (v7.0.9, LI‐COR Biosciences). Double coordinate rotation was used for tilt correction, block averaging for detrending, covariance maximisation for time lag detection and compensation for density fluctuations for the open‐path systems (Webb, Pearman, and Leuning 1980). Compensation for spectroscopic effects of temperature, pressure and water vapour were applied for the LI‐7700 measurements (McDermitt et al. 2011; Burba, Anderson, and Komissarov 2019). Raw data were filtered using Vickers and Mahrt (1997) hard flags, which screen the raw data for spikes, amplitude resolution, drop‐outs, absolute limits, skewness and kurtosis, discontinuities, time lags, angle of attack and steadiness of the horizontal wind. The fully analytical spectral corrections of Moncrieff et al. (1997) and Moncrieff et al. (2004) were applied for the high‐frequency range and low‐frequency range, respectively, for systems with open‐path analysers. High‐frequency corrections following Fratini et al. (2012) were applied at Langeweide where there is a closed‐path gas analyser.

The EddyPro output was postprocessed using a series of filters. Data was accepted if it had a quality flag of 0, indicative of the highest quality, as determined by the Mauder and Foken (2004) 0‐1‐2 system. The Licor CO_2_ and CH_4_ gas analysers have internal received signal strength indicators (RSSI), and an RSSI threshold of 70 was used for CO_2_ and 10 for CH_4_. The latter threshold of 10 was used as it usually indicates that the mirrors were dirty and/or objects were obstructing the sample path (McDermitt et al. 2011). The net ecosystem exchange of CO_2_ (NEE) and CH_4_ flux (FCH_4_) were estimated by adding the single point storage estimate to the turbulent flux (Aubinet et al. 1999). Periods at night with stable conditions, as indicated by the friction velocity (*u**), were removed using a single *u** threshold (Table 2) determined with the moving point test (Papale et al. 2006), where the threshold was determined using NEE data.

## Appendix B Biometeorological Measurements

### B.1

The project standard weather station set‐up consisted of a Gill Maximet GMX500 that recorded air temperature, relative humidity, atmospheric pressure, wind speed and wind direction, an Apogee SN500SS net radiometer (Apogee Instruments, North Logan, USA) to measure incoming and outgoing shortwave and longwave radiation, Skye SKR1840 two channel light sensor (Skye Instruments, Powys, UK) to measure photosynthetic active radiation (PPFD), Hukseflux HPF01 for the ground heat flux (Hukseflux Thermal Sensors, Delft, Netherlands), Sentek Drill and Drop soil moisture and temperature sensors (Sentek Technologies, Stepney, Australia), and Ellitrack groundwater‐level meters (Leiderdorp Instruments, Leiderdorp, Netherlands). All data were recorded using Campbell CR1000X data loggers (Campbell Scientific, Utah, USA). Meteorological data were recorded at a 1‐min frequency, soil temperature and water content at a 30‐min frequency, and groundwater levels and temperature were recorded hourly. Calibrations of soil moisture were not performed and are difficult to do in peat soils. Instead, soil moisture data were rescaled to relative per cent soil water content (SWC) using the method outlined in Nijman et al. (2024) for an indication of soil moisture conditions. The top 5 percentiles of measurements were assumed to represent saturation (100%) and the remaining values were rescaled by dividing the remaining values by the mean of the top 5 percentiles. This was done per probe and per depth increment. Reduction–oxidation (redox) potential was measured with Paleo Terra redox probes (Paleo Terra, Oijen, Netherlands) at all sites except Assendelft, Camphuys and Onlanden with the method as described in Boonman et al. (2024). The data were recorded at a 15‐min frequency. Methanogenesis is expected to occur at a thermodynamic optimum of around –140 mV at a pH of 5.5 (Varcoe et al. 2014; Boonman et al. 2024). The pH was not routinely monitored at all locations in this study, and instead of applying the linear pH correction of –59.2 mV pH for CH_4_ (Yu et al. 2007), redox potentials were normalised by setting the fifth quantile of redox potential to –200 mV. All biometeorological data were aggregated or interpolated to 30‐min timesteps to match the EC flux averaging period. If there were multiple probes of groundwater level, soil temperature and moisture or redox potential at study sites, they were averaged.

Missing meteorological data were gapfilled with data from the nearest study site. If gaps were still present, then data from the nearest Royal Netherlands Meteorological Institute (KNMI) weather station was used. The average distance to the KNMI meteorological stations was 13.7 ± 6.0 km. The data from KNMI were retrieved from the KNMI Data Platform (https://dataplatform.knmi.nl) and were aggregated from 10 min to 30 min to match the flux averaging period. Only a subset of variables was available from KNMI stations: air temperature, relative humidity, wind speed and direction, incoming shortwave radiation, pressure and vapour pressure deficit. The KNMI data were corrected using the mean bias from local station data prior to filling. Remaining missing meteorological, soil and groundwater‐level data were linearly interpolated where appropriate.

## Appendix C ML Gapfilling

### C.1

The ML gapfilling procedure will be briefly described here and a full description is available in Irvin et al. (2021). For the baseline and all predictor sets, artificial gaps were introduced into the data sets to make a single, held‐out test set, which was used for performance evaluation after model training. The artificial gap generation method considers and tries to preserve the observed distribution of gaps in the data set when creating a scoreable test set to avoid biases and skewness that can be caused by gaps in model training. Ten pairs of training and validation sets of artificial gaps were generated for each predictor set that was then used for model training. For each model and training set, a fivefold cross‐validation was used to select ML hyperparameters based on average model performance. The 10 validation sets were used to compare the algorithm classes and predictor set groups. Lastly, the 10 models of the algorithm classes that had the highest performance on the validation sets were formed into a model ensemble and the mean ensemble prediction was evaluated on the test set.
