# Supplementary material for: Drivers and Annual Totals of Methane Emissions From Dutch Peatlands
Source: Glob Chang Biol. 2024 Dec 6;30(12):e17590. doi: 10.1111/gcb.17590 (PMC11621998; doi:10.1111/gcb.17590)
Supplement: Supplementary file 1 — Data S1. Supporting Information. [file GCB-30-e17590-s003.pdf]

Supporting Information S1 to: Drivers and annual totals of methane emissions from Dutch peatlands

Study site information

Buzacott, A.J.V.[1,*], Kruijt, B.[2], Bataille, L.[2], van Giersbergen, Q.[3] Heuts, T.S. [3], Fritz, C.[3], Nouta, R.[4], Erkens, G.[5,6], Boonman, J.[1], van den Berg, M.[1] van Huissteden, J.[1,7], van der Velde, Y.[1]

Affiliation
[1] Earth and Climate, Vrije Universiteit Amsterdam, Amsterdam, Netherlands
[2] Water Systems and Global Change Group, Wageningen University, Wageningen, Netherlands
[3] Radboud Institute for Biological and Environmental Sciences, Radboud University, Nijmegen, Netherlands
[4] Wetterskip Fryslân, Leeuwarden, Netherlands
[5] Deltares Research Institute, Utrecht, Netherlands
[6] Department of Physical Geography, Utrecht University, Utrecht, Netherlands
[7] VOF Kytalyk Carbon Cycle Research, Epse, Netherlands

[*] *Corresponding author:* Alexander Buzacott (a.j.v.buzacott@vu.nl)

Contents of the file

- Table S1.1: Soil profile and chemistry information of the study sites.
- Figure S1.1 to S1.13: Mean flux footprint climatology for the study sites. A general description is provided prior to Figure S1.1.
- Table S1.2: Summary FCH_4 gap statistics by year for each site which was gapfilled
- Table S1.3: List of gaps greater than 60 days for each site

Table S1.1: Soil profiles of study sites. Please note that, when necessary, profile information was simplified. The soil profile at Zegveld was not taken within the paludiculture parcel but from a position approximately 300 m from the EC tower. The top 10 to 20 cm of soil was excavated prior to establishment of the Zegveld paludiculture site. Soil profiles were not taken at the sites De Burd (5.7 km), Hommerts (16.9 km), and Lytse Deelen (2.4 km), but two profiles from a nearby site, Aldeboarn, have been included for an indication. The distances of De Burd, Hommerts, and Lytse Deelen to Aldeboarn are 5.7 km, 16.9 km, and 2.4 km, respectively. Sediment data for the lake site, Duinigermeer, is not available.

Site	Depth [cm]	Soil class	Botanical origin	Decomposition state and plant traces	von Post	Other soil texture features	Colour	Dry BD [g cm^{-3}]	LOI [%]	C [mg C (g soil)$^{-1}$]	N [mg N (g soil)$^{-1}$]	C:N ratio	pH soil	pH porewater
Ankeveen	0-20	water	-	-	-			0.48	48.8	216.9	14.9	18.7	-	6.0
	20-35	peat	-	strongly amorphous, few root remains	H7-H10	weak sand presence	black-brown	0.94	25.0	126.7	6.5	27.1	-	5.5
	35-38	peat	-	strongly amorphous, trace of root remains	H7-H10	high sand content	black-brown	1.45	4.2	26.9	1.1	33.5	-	-
	38-70	sand	-	high humic content	-	podzol E-horizon	black-grey	0.83	27.3	122.2	8.4	24.5	-	6.2
	70-100	sand	-	moderate humic content	-	podzol B-horizon (Fe)	dark-yellow/ brown	0.57	30.7	151.7	10.9	16.3	-	6.4
Zegveld*	0-40	peat	-	strongly amorphous	H7-H10	strong clay presence	grey-brown	0.63	33.7	195.5	19.0	10.3	4.9	5.9
	40-50	peat	-	strongly amorphous	H7-H10	mild clay presence	grey-brown	0.56	46.8	325.9	25.6	12.7	4.9	5.4
	50-92	peat	wood/sedge/reed	moderately amorphous, few wood/sedge/reed	H5-H6	no mineral content	brown	0.27	66.5	358.0	23.2	15.7	5.2	5.5
	92-150	peat	wood/sedge	weakly amorphous, few wood, few sedge, some reed	H1-H4	no mineral content	brown	0.18	73.0	385.2	22.9	17.0	5.7	6.1
Camphuys	0-20	peat	sedge/typha	weakly amorphous, few sedge	H1-H4	no mineral content	dark-brown	-	-	-	-	-	-	-
	20-50	peat	sedge	moderately amorphous, few sedge, traces reed	H5-H6	no mineral content	brown	-	-	-	-	-	-	-
	50-77	peat	reed	moderately amorphous, traces sedge, few reed	H5-H6	no mineral content	black-brown	-	-	-	-	-	-	-
	77-100	wood	willow/alder	-	-	-	-	-	-	-	-	-	-	-
	100-136	peat	sedge	moderately amorphous, trace wood, few reed and sedge	H5-H6	no mineral content	brown	-	-	-	-	-	-	-
	136-150	peat	reed	weakly amorphous, traces sedge, much reed	H1-H4	no mineral content	brown	-	-	-	-	-	-	-
Ilperveld	0-10	peat	-	strongly amorphous	H7-H10	high clay content	brown-grey	0.15	83.7	397.8	17.8	23.0	5.1	-
	okt-20	peat	-	moderately amorphous	H5-H6	low clay content	grey-brown	0.26	77.3	362.8	15.1	26.3	4.8	-
	20-80	peat	sphagnum	weakly amorphous, few mosses, traces reed	H1-H4	no mineral content	red-brown	0.11	91.1	424.4	11.4	39.0	6.1	6.0
	80-90	peat	sphagnum	weakly amorphous, many mosses	H1-H4	no mineral content	yellow-brown	0.13	91.6	-	-	-	-	-
	90-150	peat	eriophorum	moderately amorphous, few heather, trace reed and mosses	H5-H6	no mineral content	red-brown	0.12	91.3	-	-	-	-	-
Onlanden	0-17	peat	-	weakly amorphous	H1-H4	no mineral content	light-brown	-	-	-	-	-	-	-
	17-31	peat	reed/sedge	moderately amorphous, traces reed and sedge	H5-H6	strong clay presence	light-brown-grey	-	-	-	-	-	-	-
	31-37	peat	-	strongly amorphous	H7-H10	no mineral content	dark-brown	-	-	-	-	-	-	-
	37-45	clay	-		-	weak silt precence	light-brown-grey	-	-	-	-	-	-	-
	45-55	peat	sedge/reed	moderately amorphous, traces sedge, few reed	H5-H6	no mineral content	dark-brown	-	-	-	-	-	-	-
	55-100	peat	sedge	weakly amorphous, few sedge, traces reed and wood	H1-H4	no mineral content	brown	-	-	-	-	-	-	-
	100-150	peat	reed	moderately amorphous, much reed, traces sedge	H5-H6	no mineral content	black-brown	-	-	-	-	-	-	-
Weerribben (floating - kragge)	0-6	peat	sphagnum	weakly amorphous, few reed, few roots	H1-H4	no mineral content	light-brown	0.03	94.7	449.5	10.6	49.5	-	-
	20-Jun	peat	sphagnum	moderately amorphous, few reed, few roots	H5-H6	weak clay presence	dark-brown	0.15	92.9	433.0	21.3	23.8	5.9	-
	20-40	peat	sphagnum	weakly amorphous, few reed, few roots	H7-H10	no mineral content	light-brown	0.22	89.4	438.0	20.3	25.2	-	-
	40-76	detritus	reed	weakly amorphous, water with litter	H1	no mineral content	yellow-brown	0.42	32.1	-	-	-	5.7	-
	76-105	peat	reed	moderately amorphous, few reed, few roots	H5-H6	no mineral content	brown	0.2	85.0	49.3	-	-	-	-
	105-150	peat	sphagnum	strongly amorphous, few reed, some reed roots	H7-H10	no mineral content	brown/dark-brown	0.43	29.9	173.4	11.1	18.0	-	-
Weerribben (land - rib)	0-20	clay	-		H1-H4	weak silt content	dark-grey	0.45	30.3	145.4	10.4	16.3	4.5	-
	20-35	peat	reed	moderately amorphous, traces sedge, few roots, few reed	H5-H6	strong clay presence	brown-grey	0.26	44.8	218.6	14.7	17.4	4.5	-
	35-60	peat	sphagnum	moderately amorphous, traces sedge, few reed	H7-H10	no mineral content	black-brown	0.29	44.3	259.7	12.5	24.4	-	-
	60-150	peat	sphagnum	weakly amorphous, some to many mosses, traces of fresh sedge	H1-H4	no mineral content	red-brown	0.1	87.4	453.4	9.0	60.3	5.4	-
Demmerik	0-30	peat	wood	strongly amorphous, traces wood	H7-H10	low mineral content, some added sand and stones	black-brown	0.49	48.9	249.4	18.5	13.5	5.3	-
	30-70	peat	wood	strongly amorphous	H7-H10	low mineral content, some added sand and stones	grey-brown	0.56	41.8	216.3	17.3	12.5	5.4	6.4
	70-90	peat	wood	strongly amorphous, traces wood	H7-H10	weak clay presence	grey-brown	0.39	59.6	-	-	-	-	6.5
	90-110	peat	wood	moderately amorphous, few wood	H5-H6	no mineral content	dark-brown	0.23	76.1	292.4	20.8	13.9	5.5	-
	110-150	peat	wood	weakly amorphous, many wood, few sedge	H1-H4	no mineral content	brown	0.16	81.5	-	-	-	-	6.4
Assendelft	0-30	peat	-	strongly amorphous	H7-H10	strong clay presence	grey-brown	0.72	29.5	147.4	12.9	11.5	5.1	
	30-40	peat	-	strongly amorphous	H7-H10	no mineral content	black-brown	0.81	19.6	-	-	-	-	
	40-140	peat	reed/sedge	moderately amorphous, few reed, few sedge	H5-H6	no mineral content	brown	0.13	86.8	338.8	19.4	17.3	5.8	
	140-150	clay						0.11	86.7	-	-	-	-	
Langeweide	0-20	clay	-	-	-	weak silt presence, strong humic content	dark-brown-grey	0.58	37.1	150.2	15.9	9.5	5.6	-
	20-40	clay	-	-	-	moderate silt presence, weak sand presence	brown-grey	0.75	21.5	-	-	-	-	-
	40-90	peat	wood	strongly amorphous, traces wood, sedge and reed	H7-H10	no mineral content	brown	0.25	65.9	302.6	23.2	12.9	5.7	6.3
	90-120	peat	wood	moderately amorphous, traces wood and sedge	H5-H6	no mineral content	brown	0.18	77.5	-	-	-	-	-
	120-130	wood (alder)						0.16	82.8	-	-	-	-	-
	130-150	peat		moderately amorphous, much wood, traces sedge	H5-H6	no mineral content	brown	0.15	67.5	-	-	-	-	-
Aldeboarn 1 (De Burd, Hommerts, Lytse Deelen)	0-50	clay	-	-	-	moderate silt presence, moderate humic content	dark-brown-grey	0.68	19.3	157.7	8.1	18.1	4.8	5.2
	50-150	peat	eriophorum	moderately amorphic, strong eriophorum presence, few sphagnum	H5-H6	no mineral content	brown	0.16	90.3	421.4	8.3	51.8	4.4	5.3
Aldeboarn 2	0-50	clay	-	-	-	weak silt presence, weak humic content	brown-grey	0.51	31.1	113.7	7.3	14.8	5.1	5.6
(De Burd, Hommerts, Lytse Deelen)	50-90	peat	eriophorum/sphagnum	moderately amorphous, few eriophorum and sphagnum	H5-H6	no mineral content	black-brown	0.16	95.4	433.9	9.6	47.5	4.2	5.2
	90-150	peat	eriophorum/sphagnum	weakly amorphous, few eriophorum and sphagnum	H1-H4	no mineral content	brown	0.12	94.7	487.8	8.2	59.8	5.1	5.9

Site aerial images and flux footprints

Each figure provides an aerial photo of the field site, the position of the eddy covariance tower, the desired flux target area shown by an orange boundary, and the mean flux footprint climatology estimated with the Kljun et al. (2015) flux footprint prediction model. Only timesteps with valid methane flux observations, that is after filtering for data quality and for the minimum contribution coming from the desired target area as shown in Table 1 in the manuscript, were used to calculate the mean flux footprint climatologies.

Zegveld

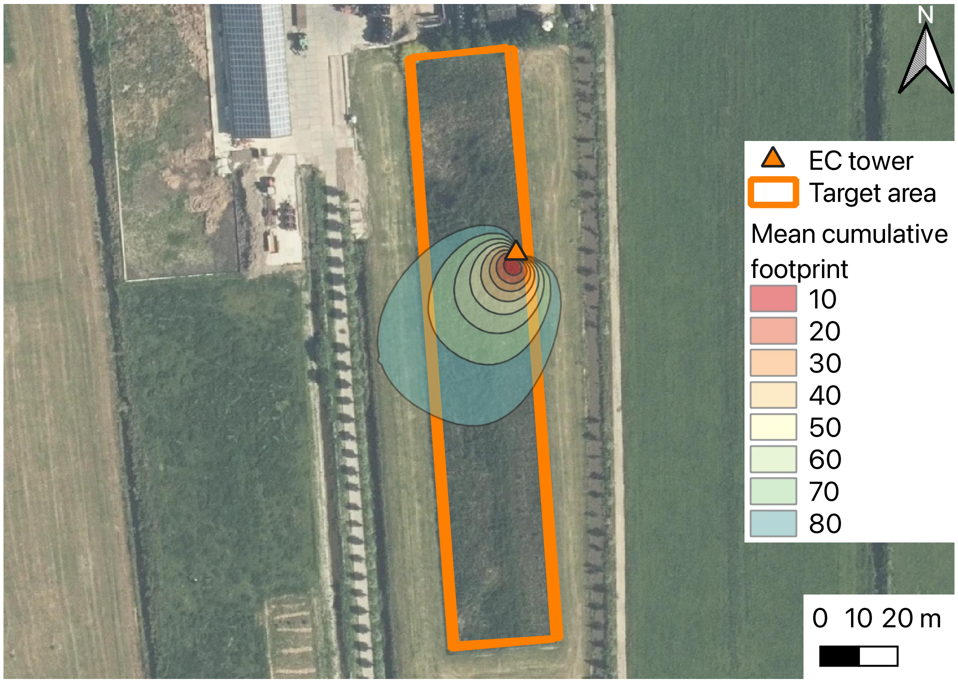

Figure S1.1: Mean flux footprint climatology for Zegveld.

Ankeveen

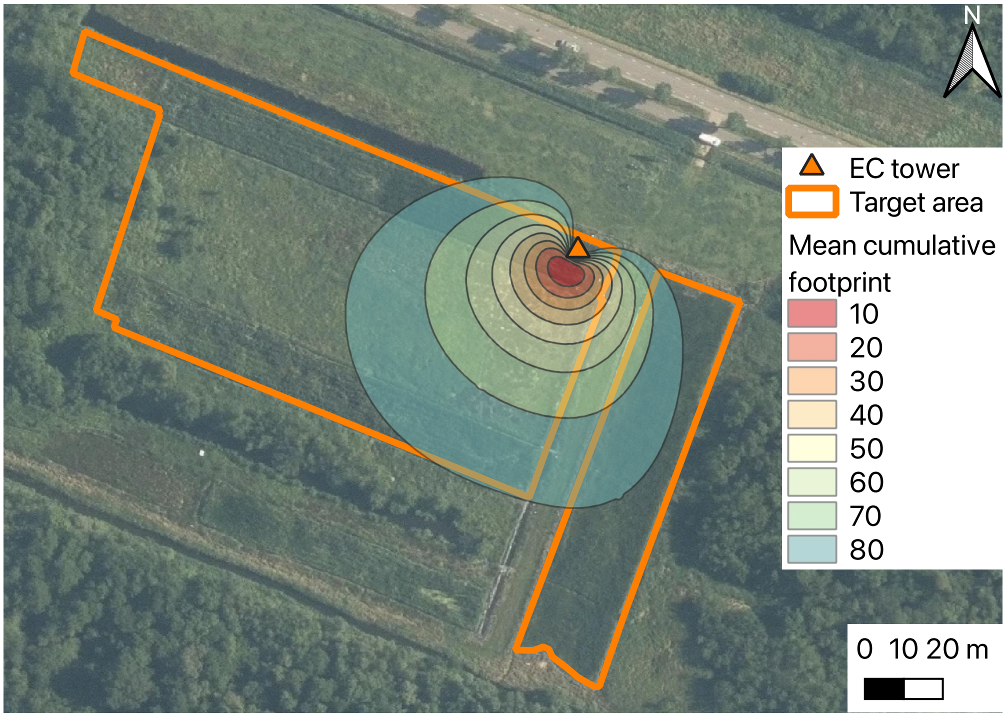

(a) Position 1: 2021-07-02 to 2023-02-11

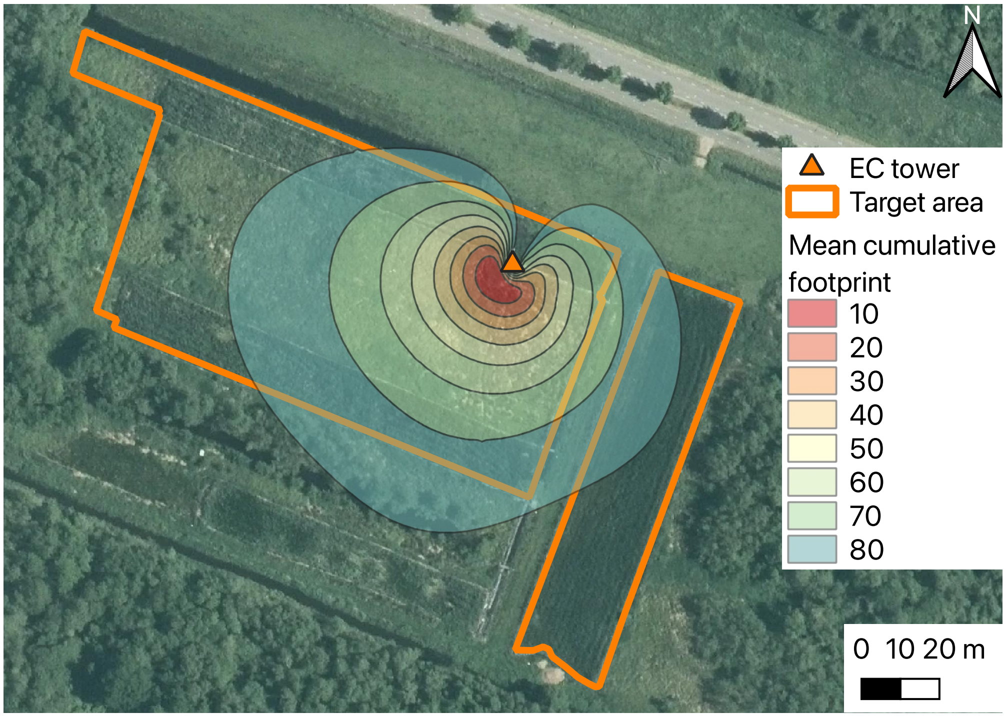

(b) Position 2: 2023-02-11 to 2023-12-31

Figure S1.2: Mean flux footprint climatology for Ankeveen. The subfigures (a) and (b) show the mean flux footprint corresponding to the two positions of the tower in the same measurement parcel.

Camphuys

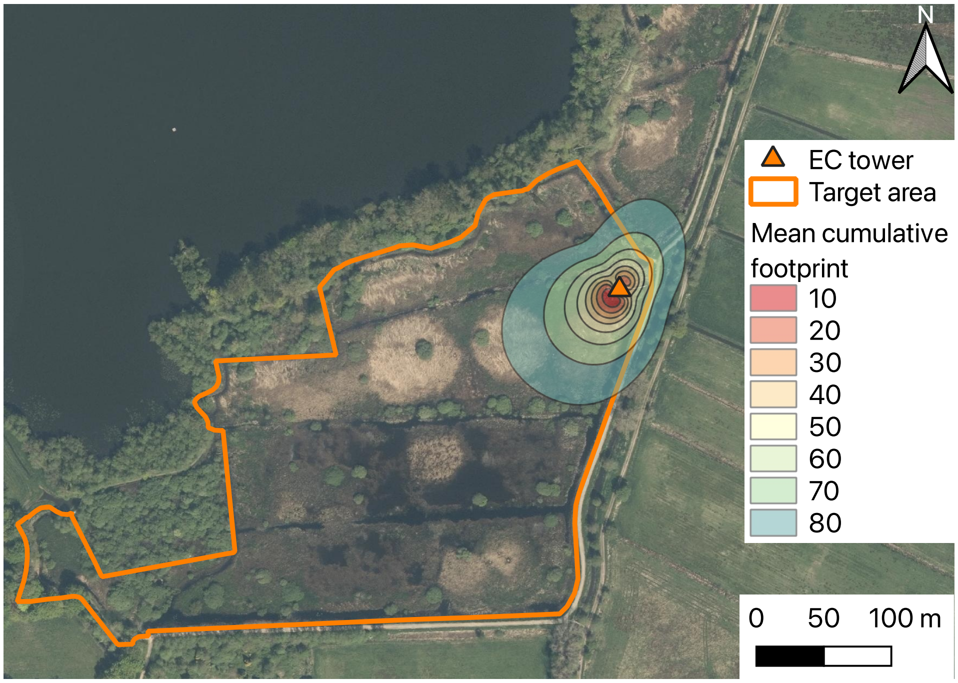

Figure S1.3: Mean flux footprint climatology for Camphuys.

Onlanden

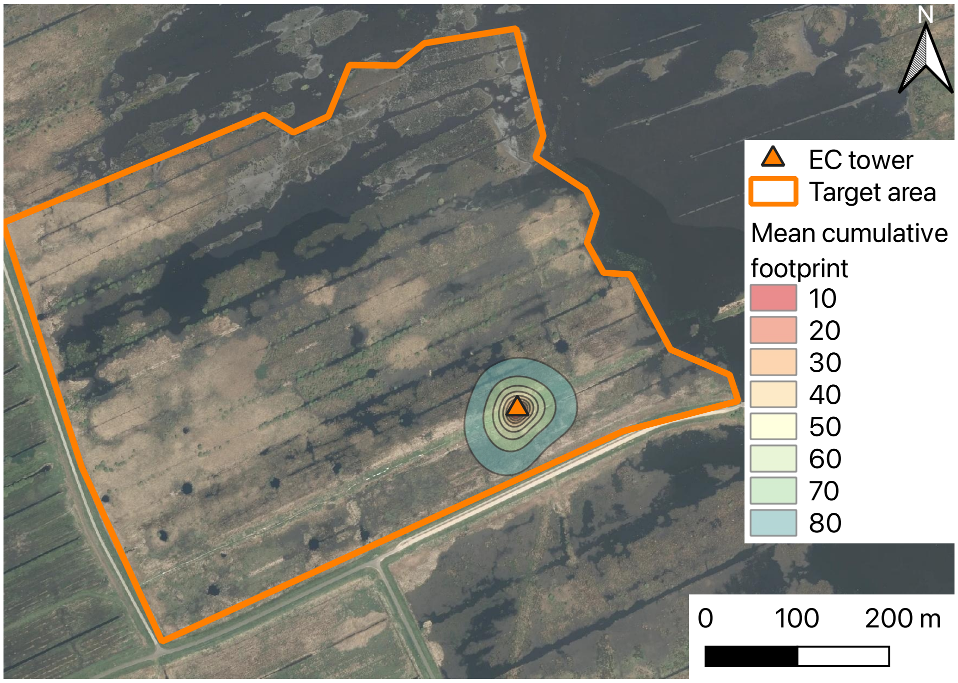

Figure S1.4: Mean flux footprint climatology for Onlanden.

Ilperveld

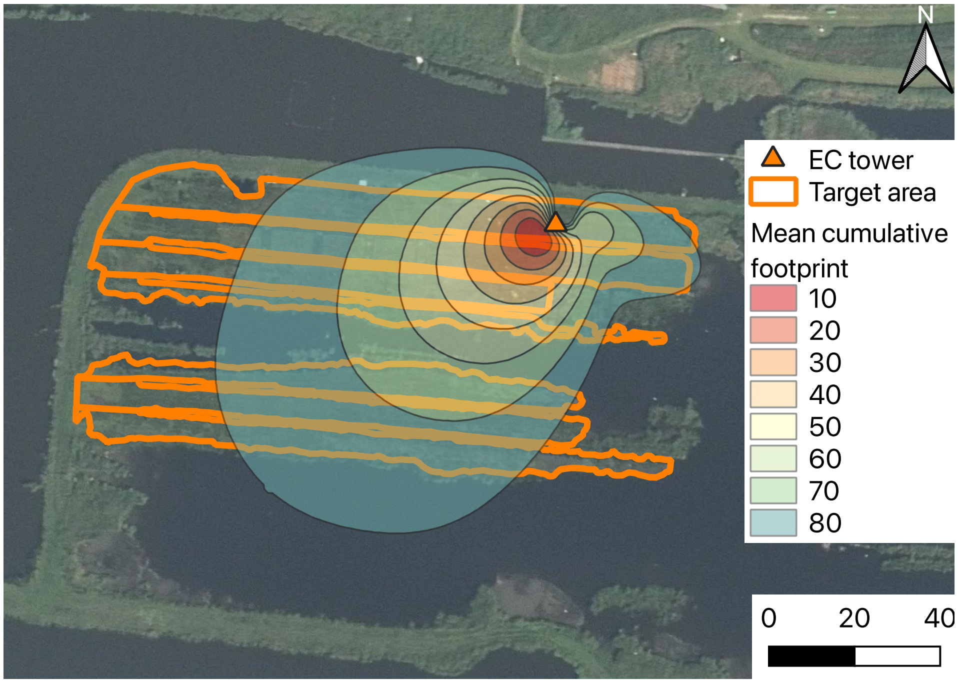

Figure S1.5: Mean flux footprint climatology for Ilperveld.

Weerribben

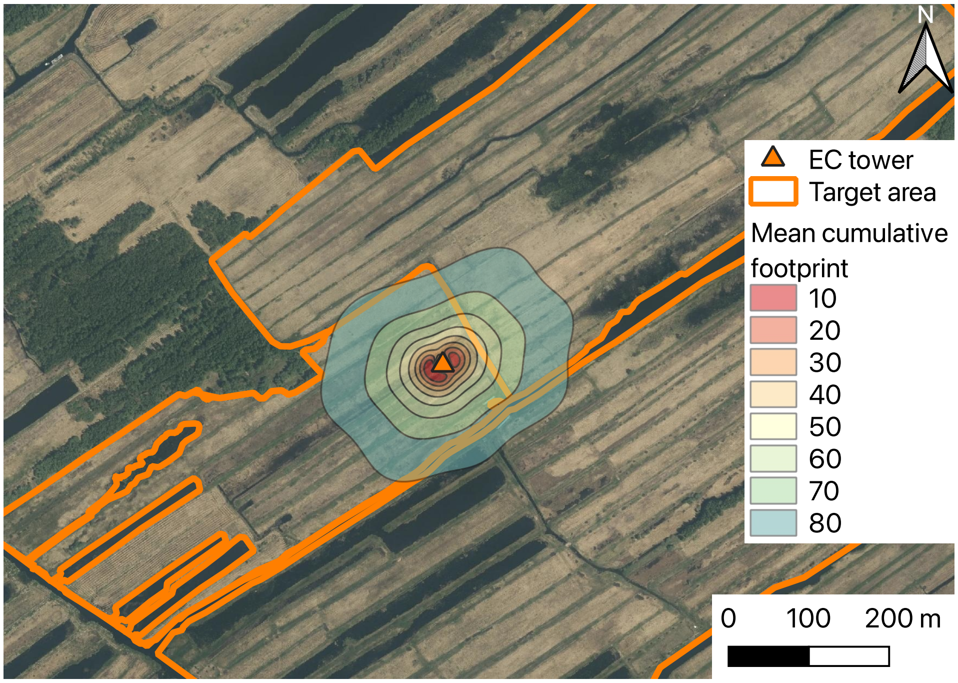

Figure S1.6: Mean flux footprint climatology for Weerribben.

Duinigermeer

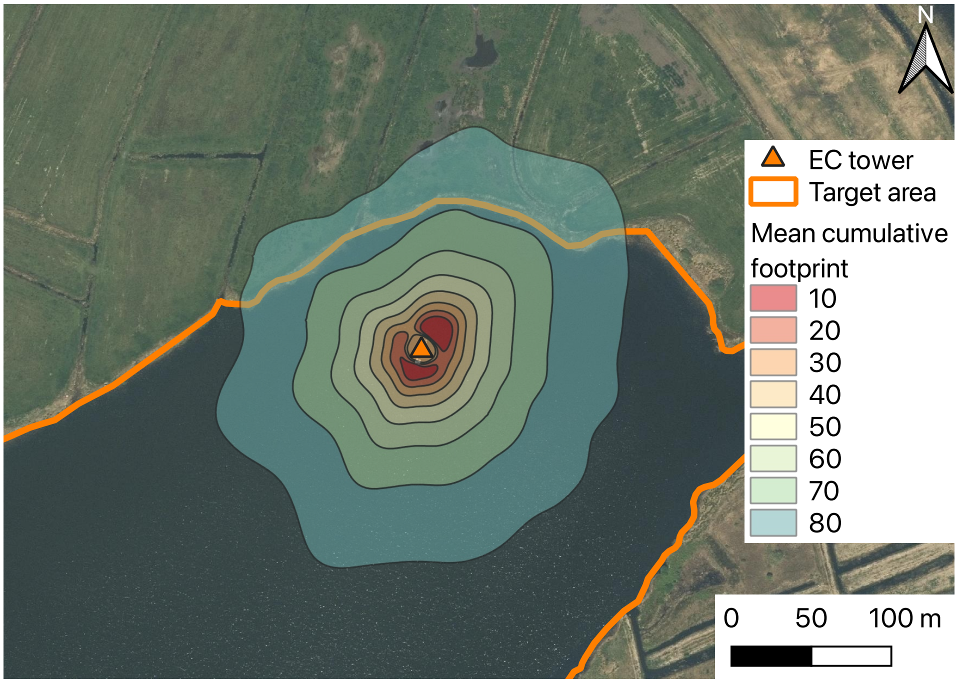

Figure S1.7: Mean flux footprint climatology for Duinigermeer.

Demmerik

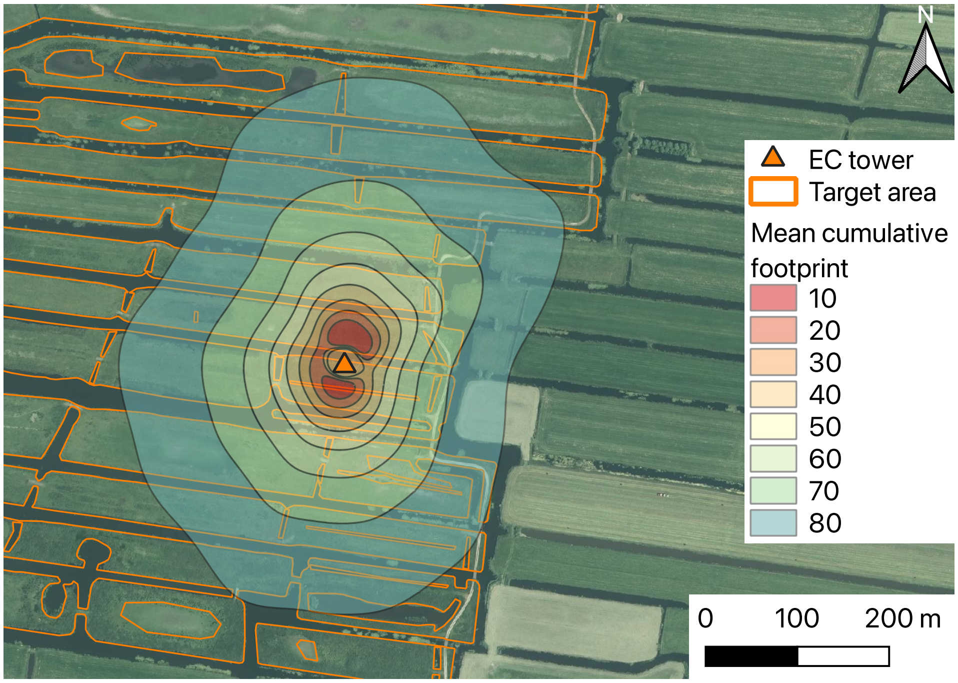

Figure S1.8: Mean flux footprint climatology for Demmerik.

Assendelft

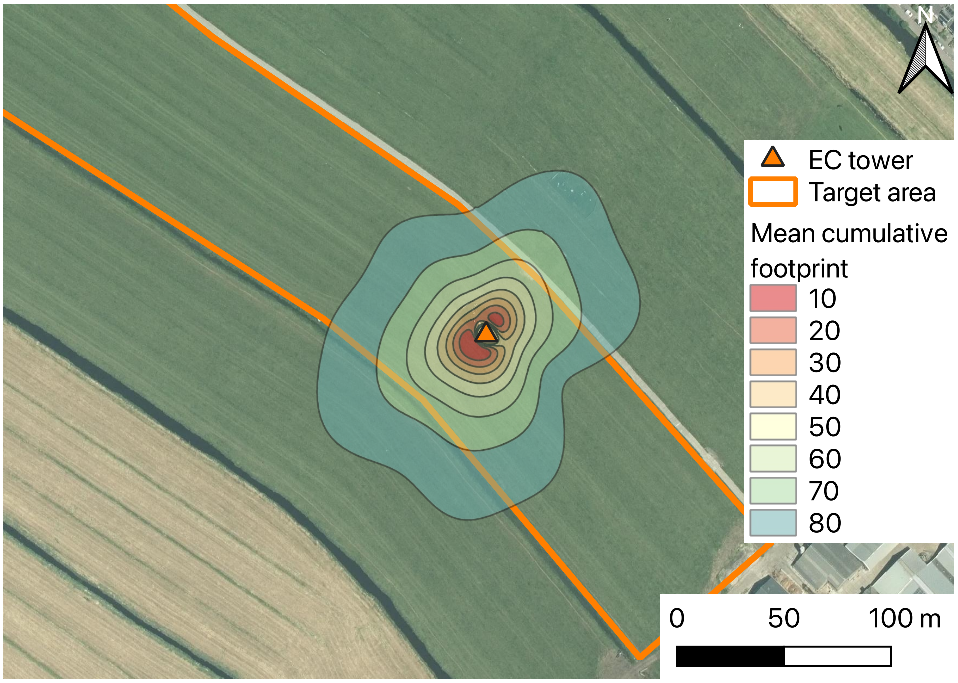

Figure S1.9: Mean flux footprint climatology for Assendelft.

Langeweide

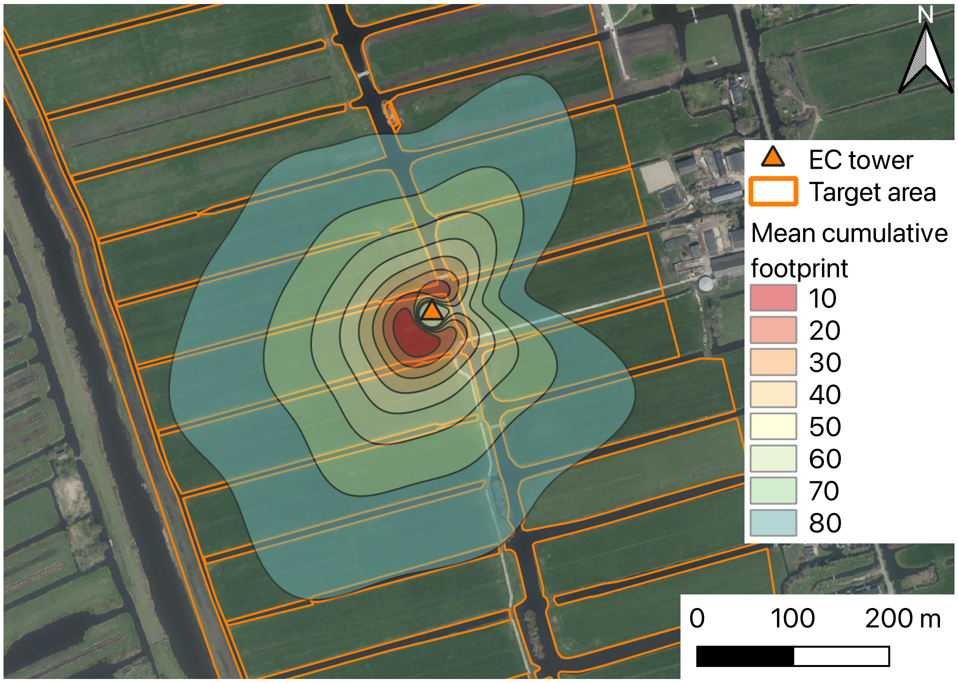

Figure S1.10: Mean flux footprint climatology for Langeweide.

Hommerts

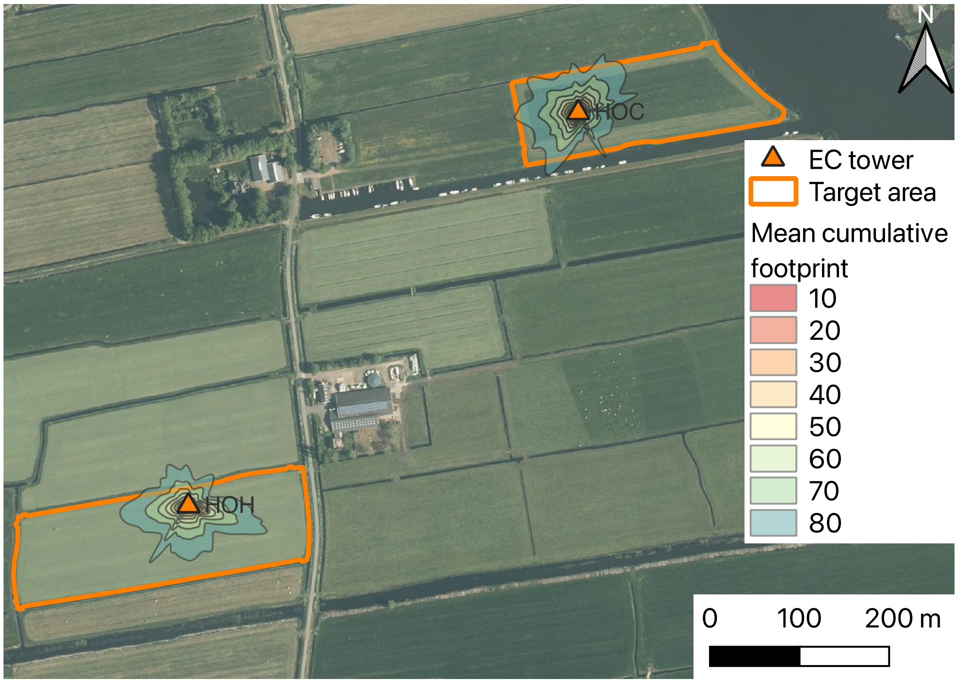

Figure S1.11: Mean flux footprint climatology for Hommerts, shown with the two treatments: control (HOC) and raised water level (HOH).

Lytse Deelen

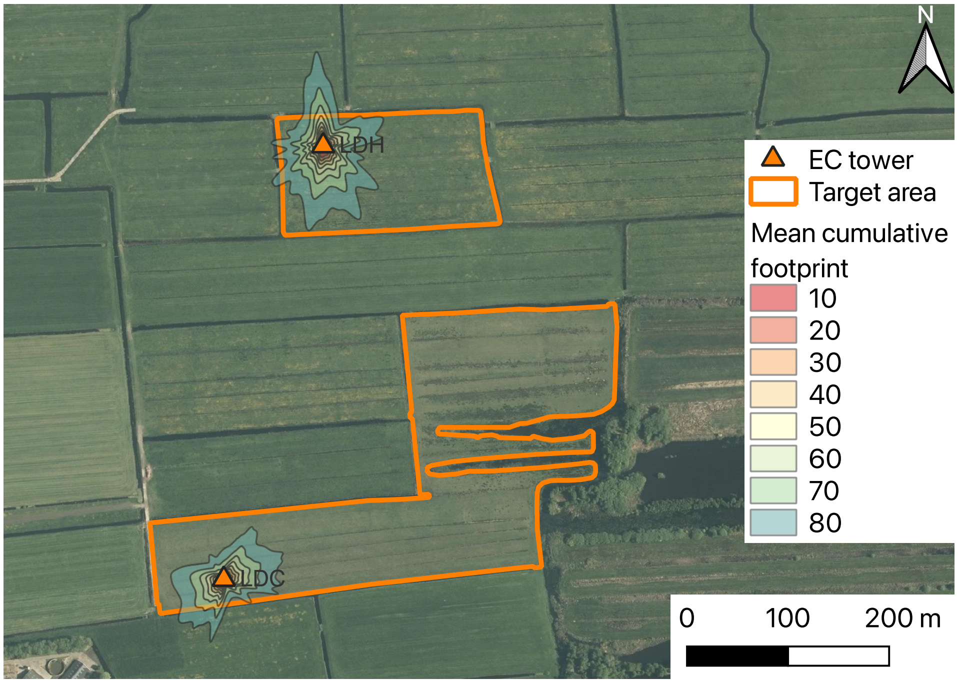

Figure S1.12: Mean flux footprint climatology for Lytse Deelen, shown with the two treatments: control (LDC) and raised water level (LDH).

De Burd

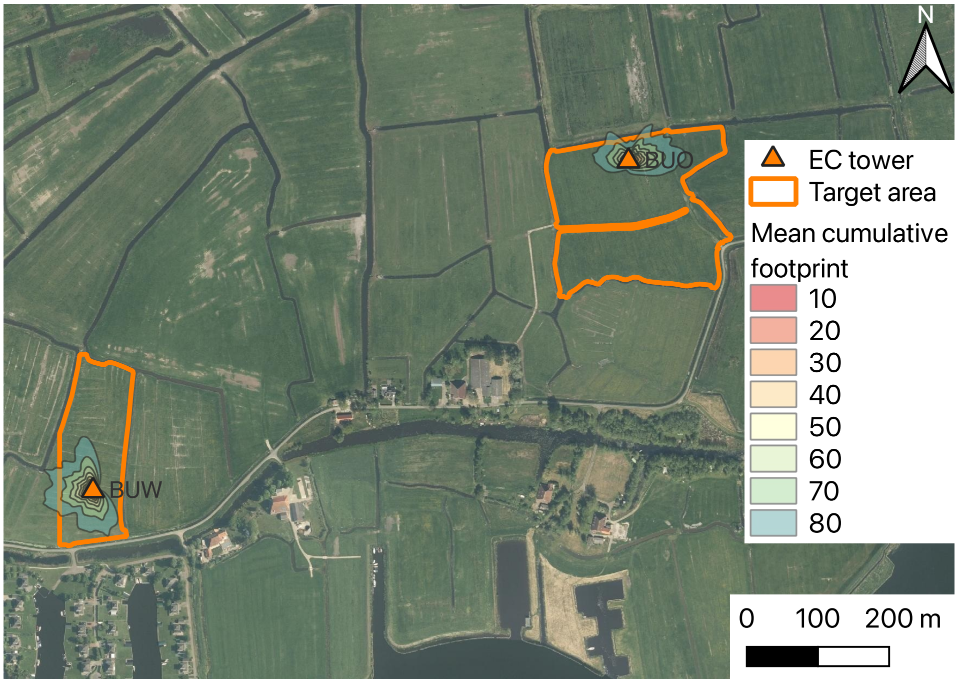

Figure S1.13: Mean flux footprint climatology for De Burd, shown with the two treatments: control (BUO) and treatment (clay cover depth) (BUW).

Table S1.2: Summary gap statistics by season for each site that was used for gapfilling. The number of data points for obsered (n obs) and gpas (n gap) refers to half-hourly fluxes. The percentage of gaps is calculated relative to the group (i.e. Gap night [%] = n gap night / n night). Seasons correspond to the northern hemisphere meteorological seasons (e.g. winter is December, January, and February).

			n		Gap [%]						
Site	Year	Date range	Obs	Gap	Total	Day	Night	Winter	Spring	Summer	Autumn
Ankeveen	2021	2021-Jul 2021-Dec	2330	6421	73	72	75	86		76	67
	2022	2022-Jan 2022-Dec	3282	14238	81	80	82	77	87	93	68
	2023	2023-Jan 2023-Dec	5332	12188	70	68	71	59	75	73	71
Zegveld	2020	2020-May 2020-Dec	574	10553	95	93	98	100	93	90	99
	2021	2021-Jan 2021-Dec	1523	15997	91	89	93	95	93	92	85
	2022	2022-Jan 2022-Dec	1351	16169	92	91	94	90	95	92	92
	2023	2023-Jan 2023-Dec	1825	15695	90	88	91	91	97	84	87
Camphuys	2020	2020-Jul 2020-Dec	606	6739	92	91	92	100		100	86
	2021	2021-Jan 2021-Dec	2934	14586	83	79	88	99	78	69	88
	2022	2022-Jan 2022-Dec	3320	14200	81	76	87	75	86	77	86
	2023	2023-Jan 2023-Oct	2048	11200	85	80	90	100	82	81	73
Ilperveld	2021	2021-Aug 2021-Dec	898	5603	86	83	89	88		87	85
	2022	2022-Jan 2022-Dec	2365	15155	87	83	90	81	84	90	92
	2023	2023-Jan 2023-Dec	2867	14653	84	80	87	83	94	74	84
Onlanden	2020	2020-Jun 2020-Dec	778	8055	91	92	91	66		100	94
	2021	2021-Jan 2021-Dec	3550	13970	80	76	84	79	77	86	78
	2022	2022-Jan 2022-Dec	2303	15217	87	83	92	95	77	84	91
	2023	2023-Jan 2023-Dec	3015	14505	83	79	87	83	83	85	80
Weerribben	2021	2021-Aug 2021-Dec	2263	4486	66	61	71	74		54	66
	2022	2022-Jan 2022-Dec	4802	12718	73	70	75	82	55	89	64
	2023	2023-Jan 2023-Dec	6844	10676	61	54	68	67	55	56	66
Duinigermeer	2021	2021-Dec 2021-Dec	0	769	100	100	100	100			
	2022	2022-Jan 2022-Dec	5625	11895	68	62	75	84	60	50	77
	2023	2023-Jan 2023-Dec	4783	12737	73	66	81	85	67	62	77
Demmerik	2022	2022-Apr 2022-Dec	2825	8936	76	67	87	100	78	56	88
	2023	2023-Jan 2023-Dec	2290	15230	87	82	93	96	85	74	93
Assendelft	2021	2021-Aug 2021-Dec	1045	6126	85	85	85	75		91	87
	2022	2022-Jan 2022-Dec	5128	12392	71	67	75	75	66	72	70
	2023	2023-Jan 2023-Dec	4612	12908	74	69	79	86	68	66	75
Langeweide	2021	2021-Nov 2021-Dec	228	1261	85	84	85	85			100
	2022	2022-Jan 2022-Dec	4164	13356	76	71	82	77	74	72	82
	2023	2023-Jan 2023-Dec	3989	13531	77	72	83	75	79	75	81
Hommerts C	2021	2021-Aug 2021-Dec	46	7205	99			100		97	100
	2022	2022-Jan 2022-Oct	215	13990	98	98	99	100	97	98	99
Hommerts H	2021	2021-Aug 2021-Dec	71	7180	99			95		100	100
	2022	2022-Jan 2022-Sep	123	12834	99			100	99	99	100
Lytse Deelen C	2021	2021-Jul 2021-Dec	82	7841	99			100		99	98
	2022	2022-Jan 2022-Oct	99	13530	99			99	100	98	100
Lytse Deelen H	2021	2021-Jul 2021-Dec	69	7854	99			100		97	100
	2022	2022-Jan 2022-Oct	257	13372	98			100	96	100	96
De Burd C	2021	2021-Jul 2021-Dec	55	7532	99			100		97	100
	2022	2022-Jan 2022-Oct	282	13011	98			100	98	98	93
De Burd T	2021	2021-Jul 2021-Dec	98	7489	99			100		97	99
	2022	2022-Jan 2022-Oct	215	13078	98			100	97	98	98

Table S1.3: Gaps longer than 60-days, their date range, and seasons for sites that were gapfilled. Multiple seasons are listed when a gap spanned acrossed seasons.

Site	Season	Date range	n days
Zegveld	SON, DJF	2020-10-07 2021-02-15	131.5
Zegveld	DJF, MAM	2022-12-29 2023-03-06	67.1
Camphuys	SON, DJF, MAM	2020-11-22 2021-03-05	102.9
Camphuys	DJF, MAM	2022-12-15 2023-03-02	76.2
Camphuys	MAM, JJA	2023-04-15 2023-06-21	66.8
Onlanden	JJA, SON	2020-07-01 2020-09-22	83.4
Onlanden	DJF, MAM	2021-12-21 2022-03-23	92.0
Demmerik	SON, DJF	2023-09-25 2023-12-02	67.7
