# Supplementary material for: Drivers and Annual Totals of Methane Emissions From Dutch Peatlands
Source: Glob Chang Biol. 2024 Dec 6;30(12):e17590. doi: 10.1111/gcb.17590 (PMC11621998; doi:10.1111/gcb.17590)
Supplement: Supplementary file 2 — Data S2. Supporting Information. [file GCB-30-e17590-s001.pdf]

Supporting Information S2 to: Drivers and annual totals of methane emissions from Dutch peatlands

Supporting results

Buzacott, A.J.V.[1,*], Kruijt, B.[2], Bataille, L.[2], van Giersbergen, Q.[3] Heuts, T.S. [3], Fritz, C.[3], Nouta, R.[4], Erkens, G.[5,6], Boonman, J.[1], van den Berg, M.[1] van Huissteden, J.[1,7], van der Velde, Y.[1]

Affiliation
[1] Earth and Climate, Vrije Universiteit Amsterdam, Amsterdam, Netherlands
[2] Water Systems and Global Change Group, Wageningen University, Wageningen, Netherlands
[3] Radboud Institute for Biological and Environmental Sciences, Radboud University, Nijmegen, Netherlands
[4] Wetterskip Fryslân, Leeuwarden, Netherlands
[5] Deltares Research Institute, Utrecht, Netherlands
[6] Department of Physical Geography, Utrecht University, Utrecht, Netherlands
[7] VOF Kytalyk Carbon Cycle Research, Epse, Netherlands

[*] *Corresponding author:* Alexander Buzacott (a.j.v.buzacott@vu.nl)

Contents of the file

- Figure S2.1: Boxplots of half-hourly methane fluxes (FCH_4) for each eddy covariance measurement site and grouped by land use.
- Figure S2.2: The diurnal cycle of methane fluxes (FCH_4). Each point is the median hourly FCH_4 for each hour per season.
- FIgure S2.3: Relationship across all land uses between daily mean groundwater level (GWL) and median methane flux (FCH_4) once the effect of temperature has been removed.
- Figure S2.4: Reduction-oxidation (redox) potential with depth and methane flux (FCH_4) across land uses where probes where available.
- Table S2.1: Spearman rank correlation of methane fluxes and redox potential with depth across land uses.

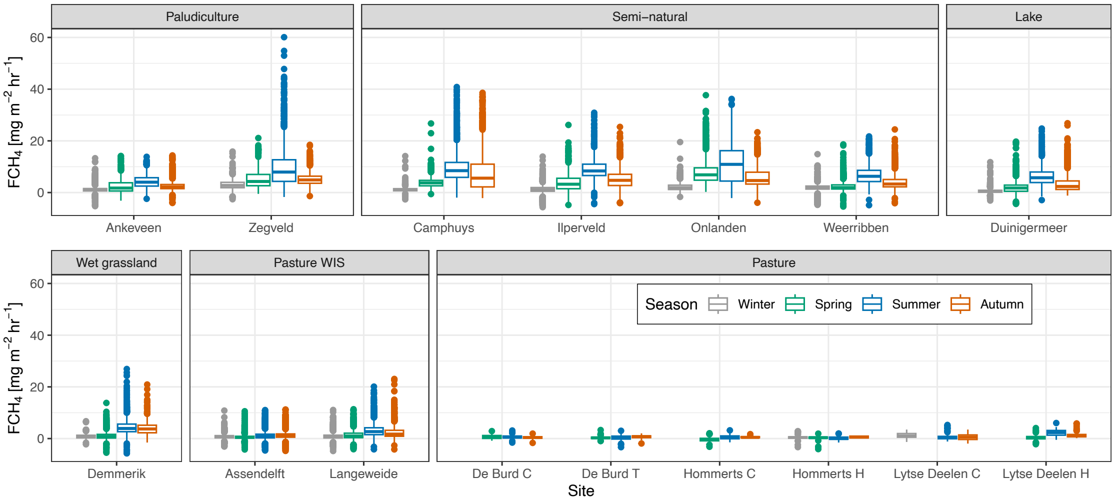

Figure S2.1: Boxplots of half-hourly methane fluxes (FCH_4) for each eddy covariance measurement site and grouped by land use. Seasons are northern hemisphere meteorological seasons. Values beyond the boxplot whiskers are included in this plot to complement Figure 3.

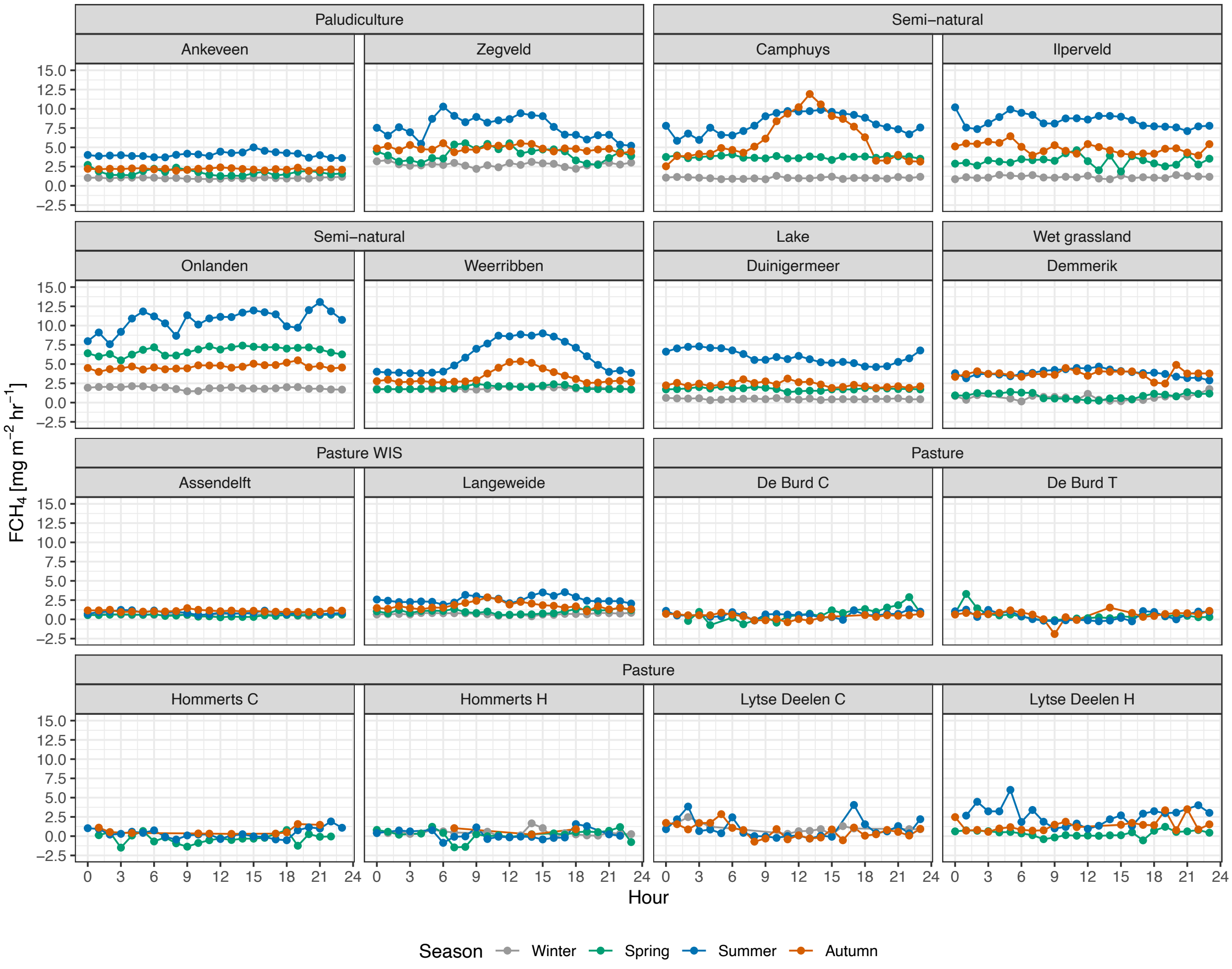

Figure S2.2: The diurnal cycle of methane fluxes (FCH_4). Each point is the median hourly FCH_4 for each hour per season.

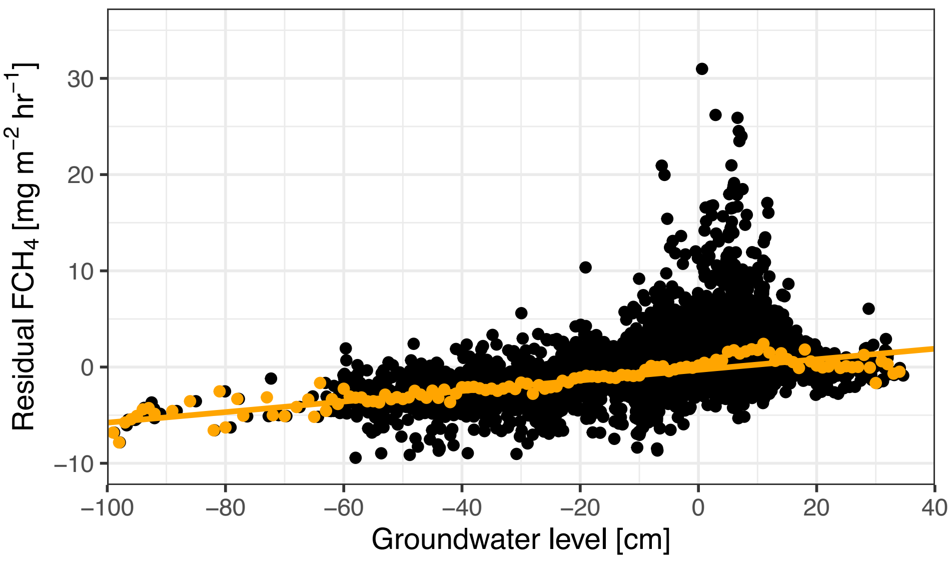

Figure S2.3: Relationship across all land uses between daily mean groundwater level (GWL) and median methane flux (FCH_4) once the effect of temperature has been removed. The temperature effect was removed by an non-linear exponential fit with the form $FCH_4 = a\exp(b\mathrm{TS})$, where TS is soil temperature. The GWL relationship was subsequently fit using the residuals of the temperature fit. To fit the GWL relationship, the data were binned in 1 cm increments and the median FCH_4 was taken for each bin (orange points), and a linear line was fit to the binned data (orange line).

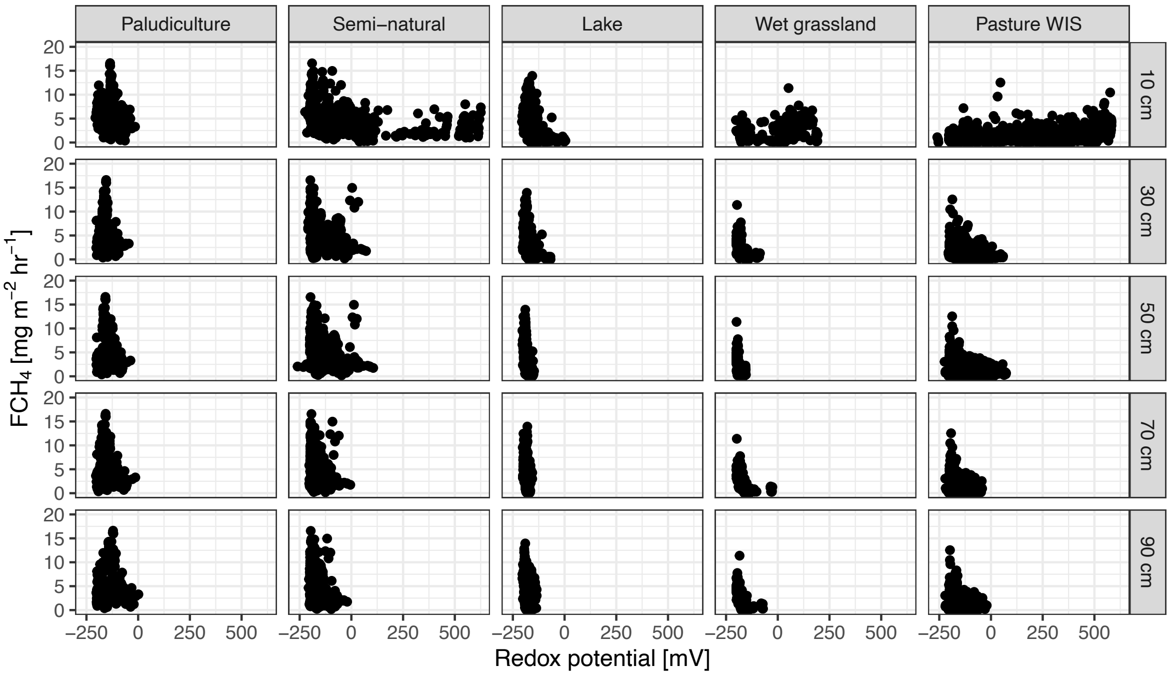

Figure S2.4: Reduction-oxidation (redox) potential with depth and methane flux (FCH_4) across land uses where probes where available. Note that the redox potential has not been linearly corrected for pH but has been standardised by setting the 5th quantile to −200 mV.

Table S2.1: Spearman rank correlation of methane fluxes and redox potential with depth across land uses. Asterisks (*) indicate variable significance at the $P < 0.05$ level.

	Redox potential				
Land use	10 cm	30 cm	50 cm	70 cm	90 cm
Paludiculture	-0.15*	-0.06	-0.19*	-0.23*	-0.20*
Semi-natural	-0.56*	-0.37*	-0.26*	-0.27*	-0.31*
Lake	-0.46*	-0.71*	-0.70*	-0.57*	-0.49*
Wet grassland	0.33*	-0.64*	-0.60*	-0.71*	-0.75*
Pasture WIS	0.31*	-0.46*	-0.32*	-0.49*	-0.43*
