# Supplementary material for: Drivers and Annual Totals of Methane Emissions From Dutch Peatlands
Source: Glob Chang Biol. 2024 Dec 6;30(12):e17590. doi: 10.1111/gcb.17590 (PMC11621998; doi:10.1111/gcb.17590)
Supplement: Supplementary file 3 — Data S3. Supporting Information. [file GCB-30-e17590-s002.pdf]

Supporting Information S3 to: Drivers and annual totals of methane emissions from Dutch peatlands

Gapfilling and annual totals material

Buzacott, A.J.V.[1,*], Kruijt, B.[2], Bataille, L.[2], van Giersbergen, Q.[3] Heuts, T.S. [3], Fritz, C.[3], Nouta, R.[4], Erkens, G.[5,6], Boonman, J.[1], van den Berg, M.[1] van Huissteden, J.[1,7], van der Velde, Y.[1]

Affiliation
[1] Earth and Climate, Vrije Universiteit Amsterdam, Amsterdam, Netherlands
[2] Water Systems and Global Change Group, Wageningen University, Wageningen, Netherlands
[3] Radboud Institute for Biological and Environmental Sciences, Radboud University, Nijmegen, Netherlands
[4] Wetterskip Fryslân, Leeuwarden, Netherlands
[5] Deltares Research Institute, Utrecht, Netherlands
[6] Department of Physical Geography, Utrecht University, Utrecht, Netherlands
[7] VOF Kytalyk Carbon Cycle Research, Epse, Netherlands

[*] *Corresponding author:* Alexander Buzacott (a.j.v.buzacott@vu.nl)

Contents of the file

- Table S3.1: Temporal coverage and predictors for each site for machine learning model gapfilling.
- Figure S3.1: Gapfilling performance on the test sets for the marginal distribution sampling (MDS) parameter combination sets.
- Figure S3.2: Gapfilling performance on the test sets for the best performing marginal distribution sampling (MDS) parameter set, lasso regression, artificial neural networks (ANN), random forest (RF), and gradient boosted regression (XGBoost).
- Figure S3.3: Mean absolute Shapley (SHAP) values for the gradient boosted regression (XGBoost) for each individual predictor in the all-predictor set.
- Table S3.2: Annual methane fluxes (FCH_4) from gapfilled timeseries using gradient boosted regression (XGBoost) machine learning (ML) models. Budgets presented are as shown in Figure 6 and Figure 7
- Table S3.3: Annual methane fluxes (FCH_4) from gapfilled timeseries from the marginal distribution sampling (MDS) and machine learning (ML) approaches Lasso regression, artificial neural networks (ANN), Random Forest (RF), and gradient boosted regression (XGB).

Table S3.1: Temporal coverage and predictors for each site for machine learning model gapfilling. The all-predictor set includes the predictors in the baseline predictors in addition to what is listed.

Land use	Site	Predictor set	Start Date	End Date	Predictors
Paludiculture	Ankeveen	Baseline	2021-07-02	2023-12-31	Temporal; Meteorological
		All	2021-10-01	2023-12-31	Baseline; H; LE; NEE; Reco; GPP; PPFD_IN; VPD; RH; P; WD; TS; SWC; REDX; WL
	Zegveld	Baseline	2020-05-14	2023-12-31	Temporal; Meteorological
		All	2021-09-01	2023-12-31	Baseline; H; LE; NEE; Reco; GPP; PPFD_IN; VPD; RH; P; WD; TS; REDX; WL
Semi-natural	Camphuys	Baseline	2020-07-31	2023-12-31	Temporal; Meteorological
		All	2020-07-31	2023-12-31	Baseline; H; LE; NEE; Reco; GPP; PPFD_IN; VPD; RH; P; WD; TS; WL
	Ilperveld	Baseline	2021-08-18	2023-12-31	Temporal; Meteorological
		All	2022-07-01	2023-12-31	Baseline; H; LE; NEE; Reco; GPP; PPFD_IN; VPD; RH; P; WD; TS; SWC; REDX; WL
	Onlanden	Baseline	2020-06-30	2023-12-31	Temporal; Meteorological
		All	2020-06-30	2023-12-31	Baseline; H; LE; NEE; Reco; GPP; PPFD_IN; VPD; RH; P; WD; TS; WL
	Weerribben	Baseline	2021-08-13	2023-12-31	Temporal; Meteorological
		All	2021-10-01	2023-12-31	Baseline; H; LE; NEE; Reco; GPP; PPFD_IN; VPD; RH; P; WD; TS; SWC; REDX
Lake	Duinigermeer	Baseline	2021-12-15	2023-12-31	Temporal; Meteorological
		All	2022-06-01	2023-12-31	Baseline; H; LE; NEE; Reco; GPP; PPFD_IN; VPD; RH; P; WD; REDX
Wet grassland	Demmerik	Baseline	2022-04-30	2023-12-31	Temporal; Meteorological
		All	2023-01-01	2023-12-31	Baseline; H; LE; NEE; Reco; GPP; PPFD_IN; VPD; RH; P; WD; TS; REDX; WL
Pasture WIS	Assendelft	Baseline	2021-08-04	2023-12-31	Temporal; Meteorological
		All	2021-08-04	2023-12-31	Baseline; H; LE; NEE; Reco; GPP; PPFD_IN; VPD; RH; P; WD; TS; SWC; REDX; WL
	Langeweide	Baseline	2021-09-30	2023-12-31	Temporal; Meteorological
		All	2022-04-23	2023-12-31	Baseline; H; LE; NEE; Reco; GPP; PPFD_IN; VPD; RH; P; WD; TS; SWC; REDX; WL

[a] Temporal predictors include day of year delta, yearly sine, yearly cosine

[b] Meteorological predictors include air temperature (TA), incoming shortwave radiation (SW_IN), air pressure (PA), and wind speed (WS)

[c] All predictor names: NEE (net ecosystem exchange), Reco (ecosystem respiration), GPP (gross primary production), PPFD_IN (incoming photosynthetically active radiation), VPD (vapour pressure deficit), RH (relative humidity), P (precipitation), WD (wind direction), TS (soil temperature), SWC (soil water content), redox potential (REDX), groundwater level (GWL)

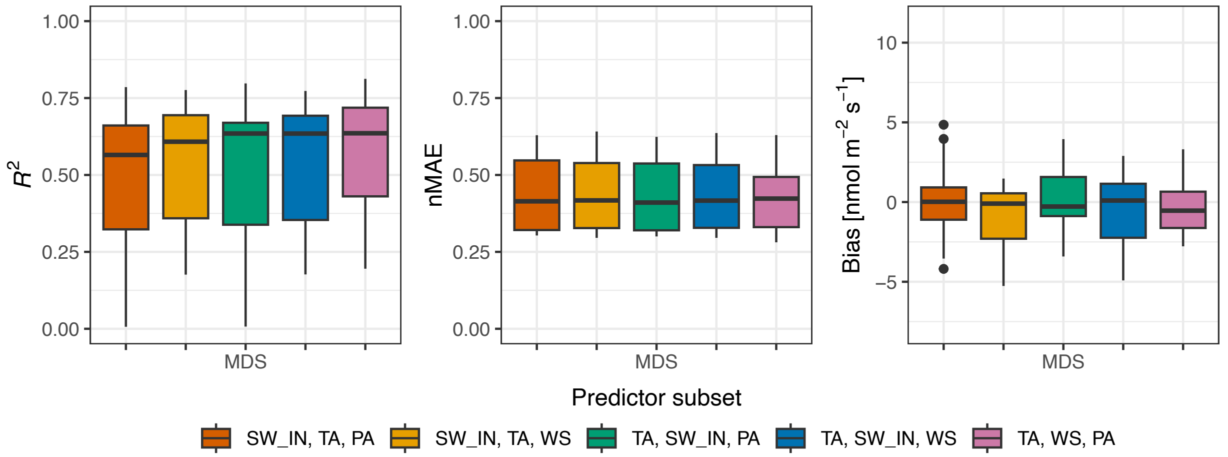

Figure S3.1: Gapfilling performance on the test sets for the marginal distribution sampling (MDS) parameter combination sets. The parameter sets included different combinations of three variables out of air temperature (TA), incoming shortwave radiation (SW_IN), air pressure (PA), and wind speed (WS).

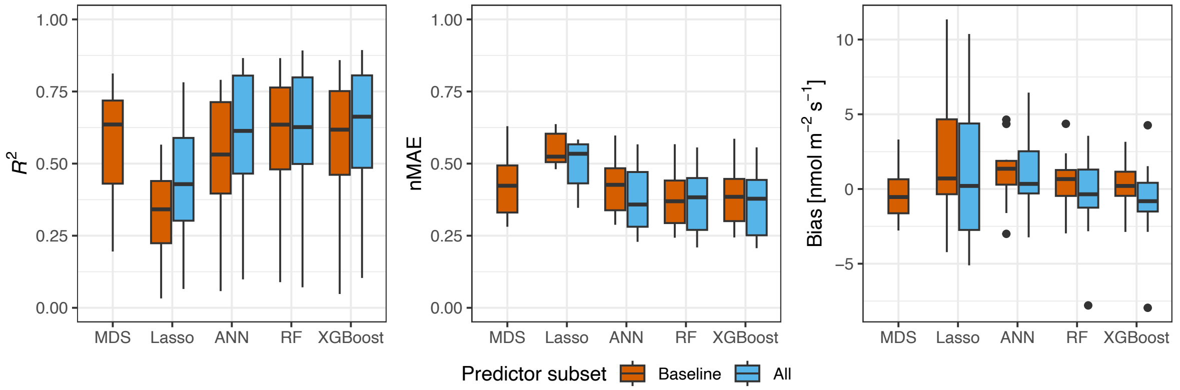

Figure S3.2: Gapfilling performance on the test sets for the best performing marginal distribution sampling (MDS) parameter set, lasso regression, artificial neural networks (ANN), random forest (RF), and gradient boosted regression (XGBoost).

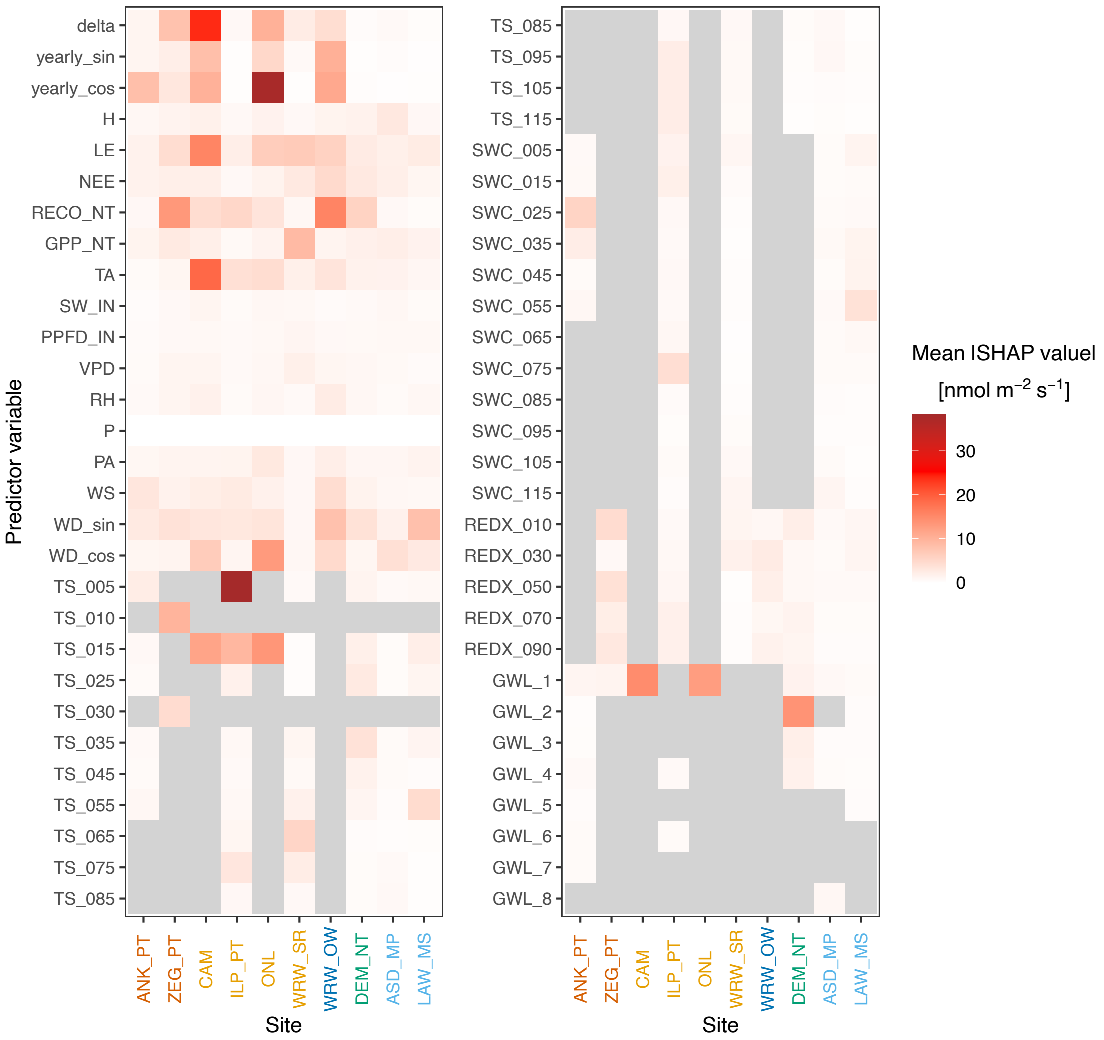

Figure S3.3: Mean absolute Shapley (SHAP) values for the gradient boosted regression (XGBoost) for each individual predictor in the all-predictor set.

Table S3.2: Annual methane fluxes (FCH_4) from gapfilled timeseries using gradient boosted regression (XGBoost) machine learning (ML) models, as shown in Figure 6. The budget with the lowest annual uncertainty from the baseline or all predictor-subsets was selected for a given site-year. The uncertainty of each budget is the 95% confidence interval from the variance of the 10 annually aggregated timeseries. The mean annual groundwater level (GWL) is provided where available.

Site	Year	Predictor subset	FCH_4 [kg CH_4 ha^{-1} yr^{-1}]	GWL [cm]
Paludiculture				
Ankeveen	2022	Baseline	306 ± 124	-7.4
	2023	Baseline	251 ± 48	-1.3
Zegveld	2021	Baseline	452 ± 111	10.5
	2022	Baseline	459 ± 145	5.9
	2023	Baseline	422 ± 123	7.4
Semi-natural				
Camphuys	2021	Baseline	320 ± 130	-6.4
	2022	Baseline	509 ± 70	3.2
Ilperveld	2022	Baseline	359 ± 77	-2.8
	2023	Baseline	402 ± 68	-3.8
Onlanden	2021	All	632 ± 65	10.2
	2022	All	411 ± 74	3.7
	2023	All	448 ± 63	10.4
Weerribben	2022	All	279 ± 32	
	2023	All	318 ± 27	-7.8
Lake				
Duinigermeer	2022	Baseline	258 ± 39	
	2023	All	259 ± 34	
Wet grassland				
Demmerik	2023	Baseline	195 ± 55	-21.0
Pasture WIS				
Assendelft	2022	All	94 ± 15	-25.0
	2023	All	90 ± 11	-22.1
Langeweide	2022	All	182 ± 20	-31.6
	2023	All	185 ± 37	-26.6

Table S3.3: Annual methane fluxes (FCH_4) from gapfilled timeseries from the marginal distribution sampling (MDS) and machine learning (ML) approaches Lasso regression, artificial neural networks (ANN), Random Forest (RF), and gradient boosted regression (XGB). The uncertainty of each budget is the 95% confidence interval (CI) from the variance of the 10 annually aggregated timeseries. The value in parentheses is the 95% CI aggregated annual residual uncertainty from the mean of the model ensembles.

		FCH_4 [kg CH_4 ha^{-1} yr^{-1}]								
			Baseline				All			
Site	Year	MDS (TA;WS;PA)	Lasso	ANN	RF	XGB	Lasso	ANN	RF	XGB
Paludiculture										
Ankeveen	2022	286 ± 3 (7)	242 ± 165 (29)	300 ± 151 (9)	300 ± 117 (5)	306 ± 124 (4)	227 ± 271 (42)	370 ± 344 (9)	308 ± 159 (5)	329 ± 139 (5)
	2023	225 ± 3 (6)	227 ± 267 (44)	233 ± 101 (8)	248 ± 94 (3)	251 ± 48 (2)	207 ± 131 (33)	308 ± 335 (7)	251 ± 114 (3)	266 ± 101 (3)
Zegveld	2021	423 ± 5 (9)	572 ± 247 (51)	490 ± 255 (25)	479 ± 112 (7)	452 ± 111 (6)				
	2022	450 ± 36 (9)	532 ± 459 (145)	468 ± 235 (39)	470 ± 143 (9)	459 ± 145 (11)	743 ± 5062 (45)	823 ± 1517 (14)	490 ± 407 (8)	481 ± 283 (7)
	2023	375 ± 14 (9)	469 ± 1139 (123)	424 ± 308 (35)	436 ± 169 (10)	422 ± 123 (10)	712 ± 4788 (52)	740 ± 1339 (16)	438 ± 317 (13)	426 ± 280 (13)
Semi-natural										
Camphuys	2021	305 ± 6 (10)	273 ± 484 (206)	312 ± 95 (25)	333 ± 63 (67)	320 ± 130 (105)	205 ± 337 (75)	320 ± 141 (20)	320 ± 60 (26)	307 ± 133 (35)
	2022	461 ± 34 (9)	489 ± 434 (71)	491 ± 253 (21)	498 ± 180 (44)	509 ± 70 (56)	532 ± 382 (89)	549 ± 169 (13)	515 ± 109 (32)	507 ± 115 (58)
Ilperveld	2022	362 ± 6 (10)	347 ± 188 (44)	356 ± 126 (28)	367 ± 189 (5)	359 ± 77 (6)				
	2023	410 ± 25 (14)	376 ± 450 (43)	393 ± 101 (32)	414 ± 55 (4)	402 ± 68 (4)	446 ± 412 (33)	453 ± 156 (11)	415 ± 105 (8)	398 ± 79 (7)
Onlanden	2021	609 ± 40 (10)	575 ± 467 (154)	618 ± 255 (48)	618 ± 96 (53)	644 ± 144 (84)	565 ± 172 (137)	604 ± 151 (19)	611 ± 89 (15)	632 ± 65 (19)
	2022	420 ± 30 (13)	533 ± 1381 (134)	447 ± 273 (72)	459 ± 190 (35)	440 ± 135 (72)	485 ± 1486 (146)	384 ± 104 (28)	433 ± 78 (28)	411 ± 74 (50)
	2023	460 ± 36 (24)	541 ± 2173 (250)	468 ± 541 (64)	459 ± 199 (136)	481 ± 194 (184)	511 ± 983 (214)	459 ± 126 (15)	448 ± 146 (63)	448 ± 63 (72)
Weerribben	2022	268 ± 4 (4)	292 ± 122 (62)	279 ± 61 (7)	280 ± 45 (3)	282 ± 50 (1)	280 ± 64 (14)	279 ± 50 (5)	279 ± 43 (2)	279 ± 32 (2)
	2023	318 ± 7 (5)	320 ± 129 (19)	318 ± 43 (7)	315 ± 40 (3)	320 ± 28 (2)	323 ± 80 (14)	325 ± 38 (5)	316 ± 32 (2)	318 ± 27 (2)
Lake										
Duinigermeer	2022	247 ± 2 (8)	263 ± 184 (57)	266 ± 85 (23)	261 ± 31 (16)	258 ± 39 (18)				
	2023	249 ± 6 (10)	264 ± 194 (71)	278 ± 82 (26)	269 ± 72 (38)	255 ± 130 (48)	267 ± 164 (42)	272 ± 31 (10)	261 ± 38 (4)	259 ± 34 (1)
Wet grassland										
Demmerik	2023	188 ± 9 (8)	205 ± 247 (23)	199 ± 167 (14)	194 ± 87 (8)	195 ± 55 (10)	196 ± 295 (13)	186 ± 420 (9)	203 ± 144 (5)	202 ± 120 (5)
Pasture WIS										
Assendelft	2022	85 ± 1 (3)	85 ± 57 (7)	88 ± 41 (6)	90 ± 34 (3)	87 ± 17 (2)	87 ± 82 (6)	97 ± 134 (4)	93 ± 39 (2)	94 ± 15 (2)
	2023	86 ± 1 (4)	85 ± 59 (8)	85 ± 31 (6)	89 ± 25 (4)	88 ± 18 (3)	87 ± 68 (7)	92 ± 89 (4)	88 ± 25 (3)	90 ± 11 (2)
Langeweide	2022	176 ± 5 (5)	170 ± 294 (24)	176 ± 71 (12)	181 ± 94 (7)	178 ± 61 (13)	185 ± 108 (9)	191 ± 63 (7)	185 ± 36 (3)	182 ± 20 (3)
	2023	174 ± 8 (6)	174 ± 238 (16)	184 ± 73 (11)	186 ± 81 (7)	183 ± 51 (8)	193 ± 118 (11)	197 ± 98 (7)	187 ± 39 (3)	185 ± 37 (3)
